# Frequent amplification of HDAC genes and efficacy of HDAC inhibitor chidamide and PD-1 blockade combination in soft tissue sarcoma

**DOI:** 10.1136/jitc-2020-001696

**Published:** 2021-02-26

**Authors:** Yi Que, Xiao-Long Zhang, Ze-Xian Liu, Jing-Jing Zhao, Qiu-Zhong Pan, Xi-Zhi Wen, Wei Xiao, Bu-Shu Xu, Dong-Chun Hong, Tian-Hui Guo, Lu-Jun Shen, Wei-Jun Fan, Huo-Ying Chen, De-Sheng Weng, Hai-Rong Xu, Peng-Hui Zhou, Yi-Zhuo Zhang, Xiao-Hui Niu, Xing Zhang

**Affiliations:** 1 Department of Medical Melanoma and Sarcoma, State Key Laboratory of Oncology in South China, Collaborative Innovation Center for Cancer Medicine, Sun Yat-sen University Cancer Center, Guangzhou, China; 2 Department of Pediatric Oncology, State Key Laboratory of Oncology in South China, Collaborative Innovation Center for Cancer Medicine, Sun Yat-sen University Cancer Center, Guangzhou, China; 3 State Key Laboratory of Oncology in South China, Collaborative Innovation Center for Cancer Medicine, Sun Yat-sen University Cancer Center, Guangzhou, China; 4 Department of Hematological Oncology, State Key Laboratory of Oncology in South China, Collaborative Innovation Center for Cancer Medicine, Sun Yat-sen University Cancer Center, Guangzhou, China; 5 Department of Minimally Invasive Interventional Therapy, State Key Laboratory of Oncology in South China, Collaborative Innovation Center for Cancer Medicine, Sun Yat-sen University Cancer Center, Guangzhou, China; 6 Department of Laboratory Medicine, The Second Affiliated Hospital of Guilin Medical University, Guilin, China; 7 Department of Orthopedic Oncology Surgery, Beijing Ji Shui Tan Hospital, Peking University, Beijing, China

**Keywords:** sarcoma, programmed cell death 1 receptor

## Abstract

**Background:**

The advent of immune checkpoint therapy has been a tremendous advance in cancer treatment. However, the responses are still insufficient in patients with soft tissue sarcoma (STS). We aimed to identify rational combinations to increase the response to immune checkpoint therapy and improve survival.

**Methods:**

Whole-exome sequencing (WES) was performed in 11 patients with liposarcoma. Somatic copy number alterations (SCNAs) were analyzed at the gene level to identify obvious amplification patterns in drug-target genes. The expression and prognostic value of class I histone deacetylases (HDACs) was evaluated in 49 patients with sarcoma in our center and confirmed in 263 sarcoma samples from The Tumor Cancer Genome Atlas (TCGA) database. Q-PCR, flow cytometry and RNA-seq were performed to determine the correlations between class I HDACs, chidamide and PD-L1 in vitro and in vivo. The efficacy of combining chidamide with PD-1 blockade was explored in an immunocompetent murine model and a small cohort of patients with advanced sarcoma. Western blot, ChIP assay and dual luciferase assessment were applied in the mechanistic study.

**Results:**

The HDAC gene family was frequently amplified in STS. SCNAs in the HDAC gene family were extensively amplified in 8 of 11 (73%) patients with liposarcoma, based on a drug-target gene set, and we verified amplification in 76.65% (197/257) of cases by analyzing TCGA sarcoma cohort. Class I HDAC expression is associated with a poor prognosis for patients with STS, and its inhibition is responsible for promoting apoptosis and upregulating of programmed cell death ligand 1 (PD-L1). The HDAC class I inhibitor chidamide significantly increases PD-L1 expression, increased the infiltration of CD8^+^ T cells and reduced the number of MDSCs in the tumor microenvironment. The combination of chidamide with an anti-PD-1 antibody significantly promotes tumor regression and improves survival in a murine model. Moreover, chidamide combined with the anti-PD-1 antibody toripalimab is effective in patients with advanced and metastatic sarcoma, and the side effects are tolerable. Mechanistically, chidamide increases histone acetylation at the PD-L1 gene through the activation of the transcriptional factor STAT1.

**Conclusions:**

The combination of chidamide and anti-programmed cell death 1 (PD-1) therapy represents a potentially important strategy for STS.

## Background

Soft tissue sarcoma (STS) represents a heterogeneous group of malignant tumors. The prognosis is poor with an overall survival of 12–19 months for patients with metastasis.^1^ More than 40% of patients diagnosed with STS in the early stage who are treated with surgery and radiation will develop metastatic disease.^2^ Therefore, a critical unmet medical need is to develop novel and effective therapeutic approaches to improve the survival of patients with STS, for whom limited alternative chemotherapeutic or targeting regimens are available.^3^

Immune checkpoint inhibitors represent new approaches in cancer treatment.^4^ PD-1 inhibitors, potentially one of the most studied inhibitors treatments, has shown clinical efficacy in various cancers, such as melanoma, non-small cell lung cancer (NSCLC) and renal cell carcinoma.^5–8^ However, anti-PD-1 antibody showed a low response rate in sarcoma. The SARC028 trial using the anti-PD-1 antibody pembrolizumab in patients with advanced sarcoma reported encouraging outcomes in patients with some specific subtypes of STS, while the total objective response rate was only 18% (7/40). In another multicenter and randomized phase II clinical trial (Alliance A091401), the confirmed overall response rate (ORR) was 5% among the 38 patients with metastatic sarcoma who received nivolumab monotherapy.^9^ Thus, the identification of a rational combination strategy to increase the response to immune checkpoint therapy remains a novel challenge.

Histone deacetylase (HDACs) mediate the post-translational acetylation of various histone proteins that act as the key mediators of gene expression modulation. Several HDAC inhibitors have been approved by the Food and Drug Administration (FDA) as treatments for hematological malignancies. SAHA (vorinostat) and romidepsin were approved as treatments for advanced cutaneous T cell lymphoma.^10^ Belinostat was approved to treat peripheral T cell lymphoma,^11^ while panobinostat was approved for the therapy of multiple myeloma.^12^ Chidamide is a novel HDAC inhibitor for the treatment of relapsed and refractory peripheral T cell lymphomas and was approved by the China Food and Drug Administration in 2014.^13^ In addition to their selective cytotoxic effects on tumor cells, HDAC inhibitors are reported to augment the efficacy checkpoint blockade therapies and regulate the host immune response.^14–16^

In the present study, we reported a copy number variation in sarcoma identified in a drug-targeted gene set using whole-exome sequencing (WES) and highlighted a potentially druggable alteration in the HDAC family genes, which has not been reported previously. We found that the mRNA expression of HDAC class I genes HDAC1, HDAC2 and HDAC3 was associated with prognosis in sarcoma. Inhibition of HDAC1/2/3 was responsible for promoting apoptosis and upregulating of PD-L1. Therefore, we hypothesized that the combination of class I HDAC inhibitors with immune checkpoint blockade in patients with sarcoma would drive an effective antitumor response. Chidamide upregulated PD-L1 expression in sarcoma in vitro and in vivo. The combination of chidamide with an anti-PD-1 antibody resulted in tumor regression and increased antitumor immune response in a murine model. We evaluated the effects of chidamide combined with an anti-PD-1 antibody named toripalimab on patients with advanced and metastatic sarcoma. Mechanistically, chidamide increased histone acetylation at the PD-L1 gene promoter and stimulated PD-L1 expression through the activation of transcriptional factor STAT1. Totally, this combination therapy showed efficacy against STS, and the side effects were tolerable.

## Materials and methods

### DNA extraction and next-generation sequencing/data processing

The methods of DNA extraction and next-generation sequencing**/**data processing are provided in the online supplemental methods.

10.1136/jitc-2020-001696.supp1 Supplementary data

### Chemicals, cell lines and patient samples

Chidamide was purchased from ApexBio. The human sarcoma cell lines were purchased from ATCC. MCA205 is a 3-methylcholanthrene-induced fibrosarcoma syngeneic to C57BL/6 mice.^17^ HT1080, SK-LMS-1, T778, RD and SW872 cells were cultured in Dulbecco's Modified Eagle Medium (DMEM) supplemented with 10% fetal bovine serum. MCA205 cells were cultured in RPMI 1640 supplemented with 10% fetal bovine serum.

Written informed consent for the collection and publication of their medical information was obtained from each patient at their first visit to our center.

### Mouse models

C57BL/6 mice were inoculated subcutaneously in the right flank with 2×10^5^ MCA205 tumor cells on day 0. Mice were then randomized into the following treatment groups: IgG control, chidamide alone, anti-PD-1 antibody alone and combination. Treatment with the drugs was started on day 5 after inoculation. The tumor volume was evaluated and calculated with the formula: (width^2^×length)/2. Chidamide was dissolved with 0.2% carboxymethyl cellulose (CMC) and 0.1% Tween 80 and administered once daily for 15 days (12.5 mg/kg/day, orally). The groups treated with out chidamide were administered using 0.2% CMC and 0.1% Tween 80 as controls. The anti-PD-1 antibody was injected through intraperitoneal injection (10 mg/kg, BioXCell) on days 6, 12, and 18 after tumor inoculation. Mice were sacrificed when the tumor’s diameter exceeded 20 mm.

### Flow cytometry analyses

Tumors were sufficiently chopped using scalpels and then digested with 0.2 mg/mL collagenase intravenous and 0.1 mg/mL DNase I at 37°C for 1 hour, and then passed through a 70 µm strainer to determine the presence of the infiltrating T cell population. Mouse-specific antibodies including anti-CD3, anti-CD4, anti-CD8, anti-CD11b, and anti-Ly6G antibodies were purchased from BD Pharmingen. Anti-IFN-γ and PD-1 antibodies were purchased from eBioscience. The human-specific antibody against PD-L1 was purchased from BD Pharmingen. Major histocompatibility complex (MHC) Class I (H-2Kb) antibody, MHC Class II (I-A/I-E) antibody, human MHC Class I/ HLA-ABC antibody (W6/32) and human MHC Class II/ HLA-DR antibody (LN3) were purchased from eBioscience. Intracellular staining for IFN-γ was performed after stimulation with PMA at 37℃ for 4–6 hours as described in the protocol of the Fixation and Permeabilization Buffer Kit (BD Bioscience). For detection of intracellular IFN-γ, brefeldin A was used to block secretion of cytokines during the last few hours of the stimulation. Cells were then subjected to flow cytometry.

### Western blotting

Total cell lysates were electrophoretically separated by sodium dodecyl sulfate polyacrylamide gel electrophoresis and transferred to a polyvinylidene difluoride membrane. Then the membrane was blocked with 5% skim milk for 1 hour at room temperature. Next, the membrane was incubated with primary antibody at 4°C overnight. Antibodies against acetylated H3K27, total H3, PD-L1, STAT1, and β-actin were purchased from Cell Signaling. Antibodies against HDAC1, HDAC2 and HDAC3 were purchased from Abcam.

## ELISPOT

An ELISPOT assay was used to detect interferon-gamma (IFN-γ) produced by CD8+ and CD4+ T cells, as previously described.^18^ MCA205 tumor cells were processed and subjected to the magnetic bead separation to isolate CD8+ T and CD4+ T cells according to the manufacturer’s recommendations (Miltenyi). Next, 10^5^/well CD8 or CD4 T cells and 5×10^4^/well of tumor cells were each plated in triplicate wells of 96-well plates and incubated at 37°C for 24 hours. MCA205 cells were stimulated with IFN-γ (20 ng/mL) for 24 hours to increase MHC expression before coculture. T cells were also cultured alone as a negative control. Spot detection was assessed using a mouse IFN-γ precoated ELISPOT kit (Dakewe Biotech Co, Ltd). Spot counting was performed with an AID ELISPOT Reader System.

### Quantitative RT-PCR

Total RNA was extracted using TRIzol reagent (Invitrogen) according to a standard protocol, and the concentration of RNA was determined. Then, cDNAs were synthesized with a reverse transcriptase kit (Invitrogen). Expression was assessed using a qPCR analysis with SYBR Green on a Bio-Rad CFX96 PCR instrument. The resulting data were assessed, and relative mRNA expression was calculated with the formula 2[−(delta delta Ct)]. Primers are listed in online supplemental table S2.

### HDAC gene amplification analysis using quantitative-PCR (q-PCR)

*HDAC1, HDAC2 and HDAC3* amplification were analyzed by using q-PCR with the method described by Ma and Chung.^19^ The level of amplification of *HDAC* genes were calculated as described by Lee *et al*.^20^ Positive amplification was considered when the copy number was greater than two times that of the reference gene. Primers are listed in online supplemental table S3.

### ChIP assay

ChIP assays were performed as described by Zeng *et al*.^21^ Briefly, 5×10^6^ HT-1080 cells were treated with or without chidamide for 24 hours. A total of 5 µg primary antibodies against acetylated-histone H3K27 from Cell Signaling and IgG control from R&D Systems were used for each immunoprecipitation. Protein A beads were incubated with the reactions for 2 hours at 4℃. Then, the beads were washed and eluted, and a PCR Purification Kit (Qiagen) was used to purify DNA. DNA was subjected to a q-PCR analysis using Bio-Rad CFX96 PCR instrument. PD-L1 promoter primers are listed in online supplemental table S4.

### Dual-luciferase reporter assay

HT-1080 cells were transfected with the STAT1-targeted siRNA or siRNA NC overnight and then cotransfected with the pGL3-basic vector or the PD-L1 promoter luciferase reporter gene plasmid and the pRL-TK plasmid for 48 hours. Chidamide or dimethylsulfoxide (DMSO) was added for another 24 hours. Cell lysates were harvested for the dual-luciferase assay, which was performed according to the manufacturer’s instructions (Promega, Madison, Wisconsin, USA). Data were normalized to Renilla luciferase activity.

### RNA-seq

Cells were treated with chidamide or the DMSO control for 48 hours. Total RNA was isolated using TRIzol reagent. Library construction was performed with the generated 100 bp paired-end reads. Then the libraries were sequenced using the Illumina HiSeq 2500 platform by Annoroad Gene Technology (Beijing, China).

### Cell proliferation assay

Sarcoma cells were seeded in 96-well plates and incubated overnight. Then, cells were treated with the control or various concentrations of chidamide for 0–5 days. Add 20 µL of 0.5 mg/mL 3-(4,5-Dimethylthiazol-2-yl)−2,5- diphenyltetrazolium-bromide (MTT) solusion into each well and incubate the plates at 37°C for 4 hours. Then, the media were carefully discarded, and the cells in each well were lysed with 200 µL of DMSO. The absorbance was measured at optical density (OD)=490 nm within 1 hour.

### Colony formation assay

Colony formation assays were performed as described by Yuan *et al*.^22^ Briefly, cells were plated in six-well plates and cultured with DMEM in the presence or absence of chidamide for 14 days. Then the colonies were fixed with methanol and stained with 0.5% crystal violet. Three independent wells were established for each chidamide treatment concentration.

### RNA interference assay

Sarcoma cells were transfected with siRNAs using Lipofectamine RNAiMAX Reagent (Invitrogen, Waltham, Massachusetts, USA) according to the manufacturer’s instructions.

### Statistical analysis

Overal survival (OS) and disease-free survival (DFS) were analyzed using Kaplan-Meier and log rank tests. The significance of difference in expression in different groups was determined by an unpaired, two-tailed t-test or one-way analysis of variance. Statistical analyzes were performed using GraphPad Prism V.6.0 software and SPSS V.19.0. Statistical significance was defined as p<0.05.

## Results

### Extensive amplification of the *HDAC* gene family in liposarcoma

We recruited 11 Chinese patients with pathologically confirmed liposarcoma and performed WES of the tumor–blood sample pairs from these patients. In this cohort, we detected 328 (mean 29.82) somatic non-silent mutations in 306 genes. TP53, which was previously reported to be the most recurrently mutated gene in sarcomas,^23^ was recurrently mutated in these patients (online supplemental figure S1).

**Figure 1:**
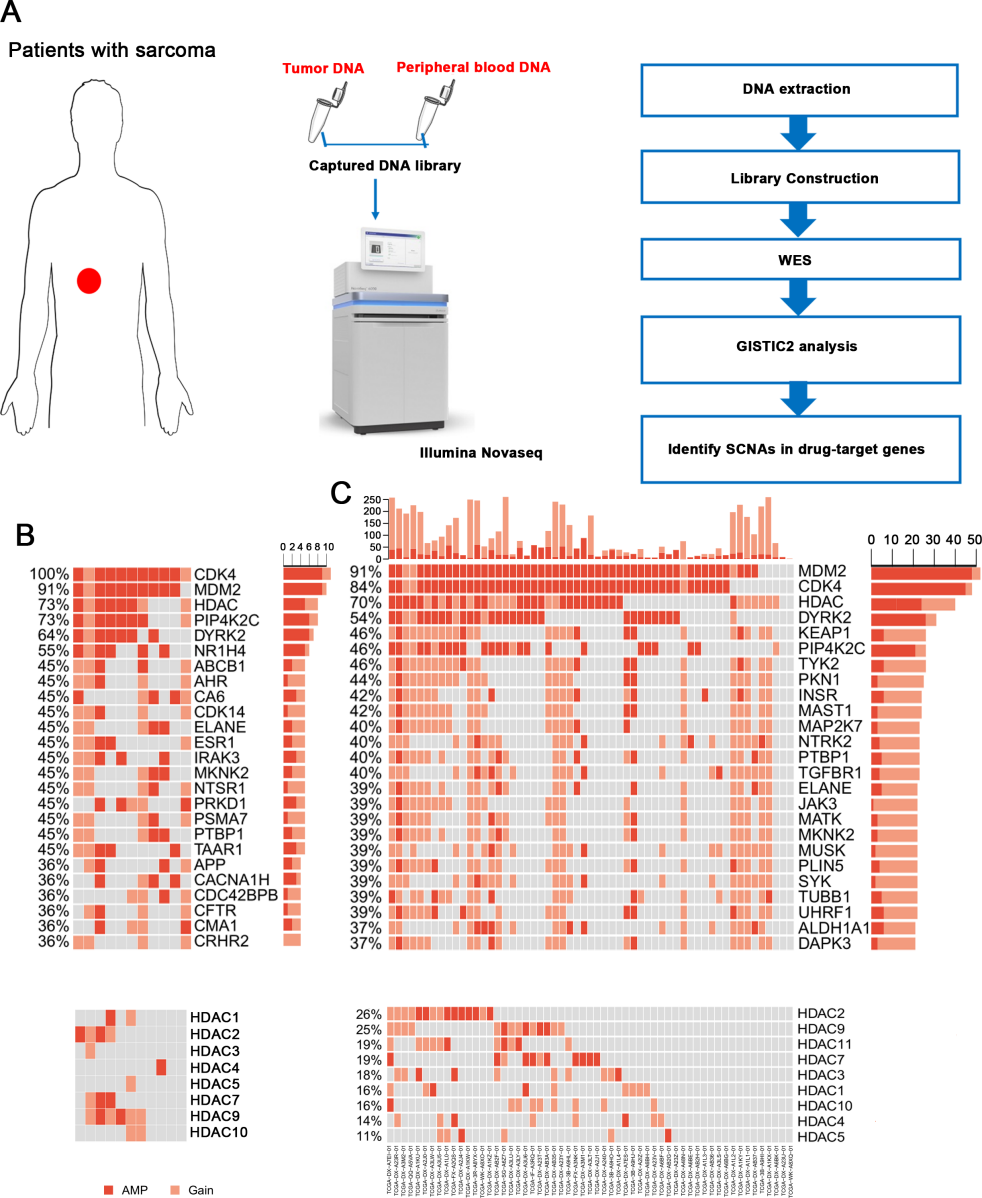
Gene amplification patterns of drug-target genes. (A) The procedures of WES and data analysis. Tissue and peripheral blood DNA of patients with sarcoma were extracted. Exon libraries were constructed using capture kits, and WES was performed. Then data were analyzed using GISTIC2. (B) Somatic amplifications/gains of drug-targeted genes/gene families (upper). HDAC gene family was extensively amplified in sarcoma (lower). (C) Somatic amplifications/gains of drug-targeted genes/gene families in TCGA liposarcoma samples (upper). (D) HDAC gene family was extensively amplified in TCGA liposarcoma samples (lower). HDAC, histone deacetylase; SCNA, somatic copy number alteration; TCGA, The Cancer Genome Atlas; WES, whole-exome sequencing.

Then, we identified somatic copy number alterations (SCNAs) and detected significant large segment copy number gains, including gains at chromosomes 6q24.3, 12p13.31 and 12q14.1 (online supplemental figure S2). The gains at chromosome 12q13~15 were previously reported as highly recurrent focal amplifications in all subtypes of sarcoma, and in our patients, the SCNA peak at 12q14.1 was the most significant amplification. Because gene amplification is a common basis for resistance to anticancer drugs, we analyzed SCNAs at the gene level and tried to identify obvious amplification patterns in drug-target genes (figure 1A). As expected, we detected *CDK4* and *MDM2* amplification in all samples and 10 of 11 samples, respectively. The expression of these two genes has been reported in well and dedifferentiated liposarcoma.^24^ CDK4 inhibitors, such as palbociclib, are FDA approved for breast cancer therapies,^25^ and MDM2 inhibitors, including nutlin-3, also display exciting prospects.^26^ Interestingly, we found that the *HDAC* gene family was also extensively amplified in 73% of the samples (*HDAC1* in 2/11 patients, *HDAC2* in 4/11, *HDAC3* in 1/11, *HDAC4* in 1/11, *HDAC5* in 1/11, *HDAC7* in 3/11, *HDAC9* in 6/11 and *HDAC10* in 2/11 patients; figure 1B). These genes were also idendified as frequently amplified in The Cancer Genome Atlas (TCGA) liposarcoma cohort (figure 1C). Furthermore, the *HDAC* gene family was extensively amplified in 76.65% (197/257) of all sarcoma samples with different subtypes in TCGA cohort and were particularly amplified in fibrosarcoma (22/24, 91.67%), undifferentiated sarcoma (34/34, 100%) and leiomyosarcoma samples (76/101, 75.25%) (online supplemental figure S3). Based on this finding, HDAC inhibitors may be potentially effective drugs for sarcoma treatment.

### Class I HDAC expression is associated with a poor prognosis for patients with STS

**Figure 2:**
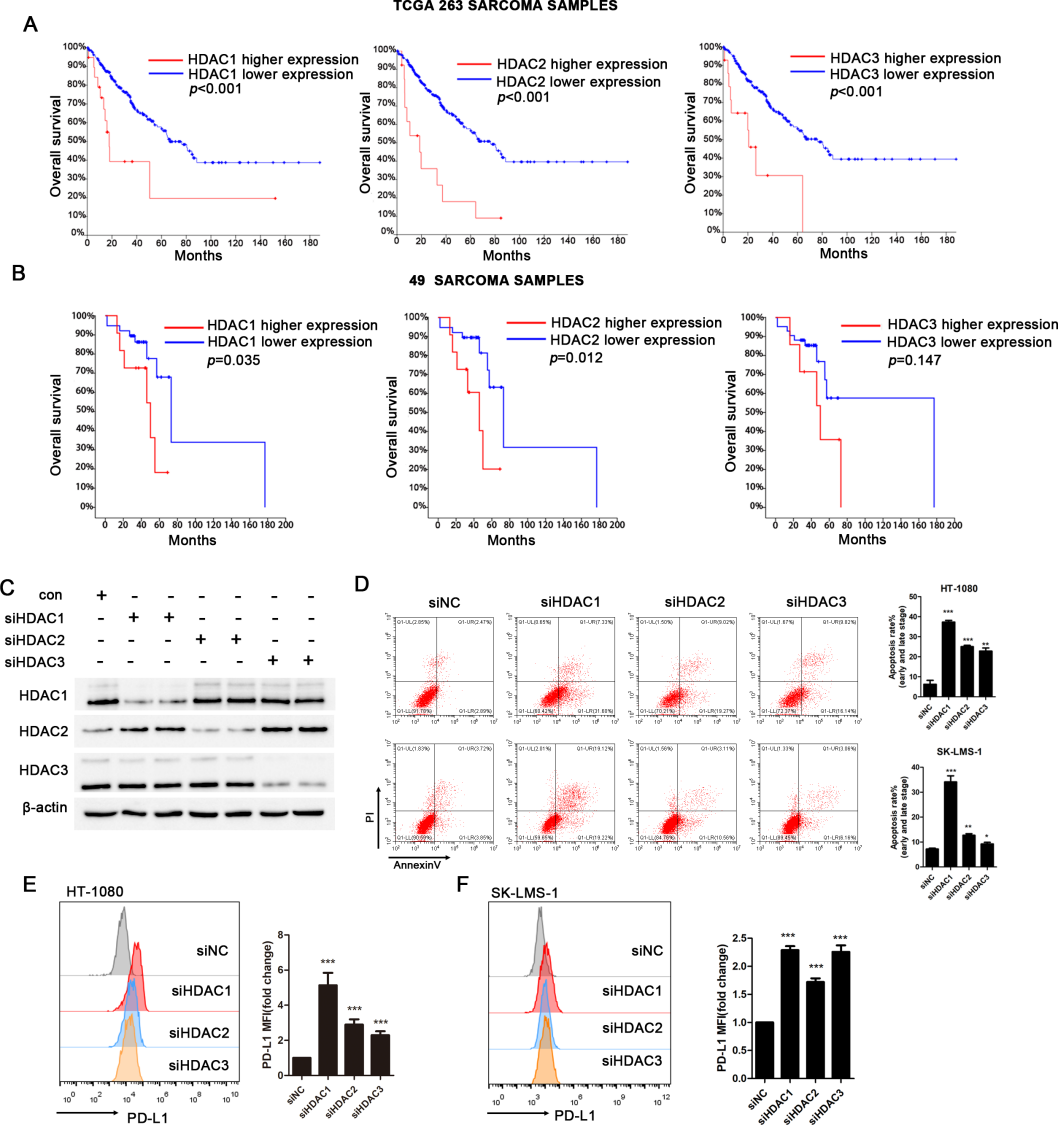
Prognostic significance of HDAC1, HDAC2, and HDAC3 expression in STS patients. (A) Prognostic significance of HDAC1, HDAC2, HDAC3 mRNA expression in 263 TCGA sarcoma samples. HDAC1, HDAC2, and HDAC3 mRNA upregulation is significantly correlated with poor overall survival (p<0.001); the mRNA expression levels calculated by RSEM (TCGA V2) were scaled as z-scores with threshold ±2.0. (B) Prognostic significance of HDAC1, HDAC2, and HDAC3 mRNA expression in 49 patients with STS. The mRNA expression in STS was detected using real-time PCR. Kaplan-Meier and log rank analyses for overall survival of STS patients with available clinical follow-up data identified two subgroups with higher or lower expression. HDAC1 and HDAC2 mRNA expression is significantly associated with overall survival (p=0.035 and p=0.012, respectively), and HDAC3 is not much significantly associated with overall survival (p=0.147). (C) Western blot analysis of whole cell lysates derived from HT-1080 cells after class I type HDACs siRNA transfection. (D) The apoptosis rate of HT-1080 and SK-LMS-1 after transfected with class I type HDACs (HDAC1, HDAC2 and HDAC3). (E-F) Flow cytometry analysis of surface levels of PD-L1 on class I type HDACs (HDAC1, HDAC2 and HDAC3) knockdown HT-1080 cells (left) and SK-LMS-1 cells (right) and quantification. Data are mean±SD of three independent experiments. *P<0.01 (two-tailed unpaired t-test). HDAC, histone deacetylase; STS, soft tissue sarcoma; TCGA, The Cancer Genome Atlas.

We analyzed the TCGA public database of 263 STS samples (cBioPortal.org) to identify the potential role of HDACs in sarcoma and observed an association between higher expression of class I HDACs, including HDAC1, HDAC2 and HDAC3 with shorter overall survival (p<0.001) (figure 2A). We also performed real-time PCR (RT-PCR) with cDNAs from human STS samples and analyzed the clinical significance of class I HDAC expression including HDAC1, HDAC2 and HDAC3, in 49 patients with different subtypes of STS treated at our cancer center. Patients with higher HDAC1 and HDAC2 mRNA expression exhibited a significantly lower overall survival rate (p<0.05). A trend, although not significant, toward shorter overall survival was observed for patients with higher expression of the HDAC3 mRNA (figure 2B). The characteristics of these 49 patients with sarcoma are shown in online supplemental table S1. In particular, patients with liposarcoma and fibrosarcoma patients accounted for 40.8% of all the patients evaluated (10 patients with liposarcoma and 10 patients with fibrosarcoma).

Then we used siRNAs to knockdown class I HDACs, respectively (figure 2C). As shown in figure 2D, siRNA-mediated knockdown of class I HDACs (HDAC1, HDAC2, and HDAC3) significantly promoted cell apoptosis. At 48 hours after siRNA transfection, knockdown of HDAC1 resulted in the highest apoptosis rate including early and late apoptosis stages. Moreover, knockdown of class I HDAC genes was responsible for the upregulation of PD-L1 in sarcoma cells (figure 2E–F).

Thus, class I HDAC expression is associated with a poor prognosis, and the inhibiton of class I HDAC expression induces apoptosis and increases PD-L1 expression in sarcoma.

### The class I HDAC inhibitor chidamide increases histone acetylation and exerts potential inhibitory effects on sarcoma cell lines

Because class I HDAC expression indicated a poor prognosis and chidamide is a potential HDAC inhibitor targeting class I HDACs, we detected the effects of chidamide on sarcoma cells and used the upregulation of acetylated histone H3K27 as a marker of class I type HDAC inhibition. HT-1080, SK-LMS-1 and T778 sarcoma cells were treated with chidamide at the indicated doses. As shown in online supplemental figure S4A, chidamide increased the acetylation of H3K27. Since class I HDACs affect cell proliferation,^27^ we also detected the expression of HDAC1, HDAC2, and HDAC3 after treatment with chidamide and founded inhibited expression of HDAC1, HDAC2, and HDAC3 in the three sarcoma cell lines (online supplemental figure S4B). MTT assays and colony formation assays were performed in cells treated with various concentration of chidamide to examine the effect of chidamide on the proliferation of sarcoma cell lines (online supplemental figure S4C-F). Chidamide suppressed the proliferation of sarcoma cells in a dose-dependent manner.

### Chidamide upregulates MHC class I genes and PD-L1 expression in sarcoma in vitro and in vivo

**Figure 3:**
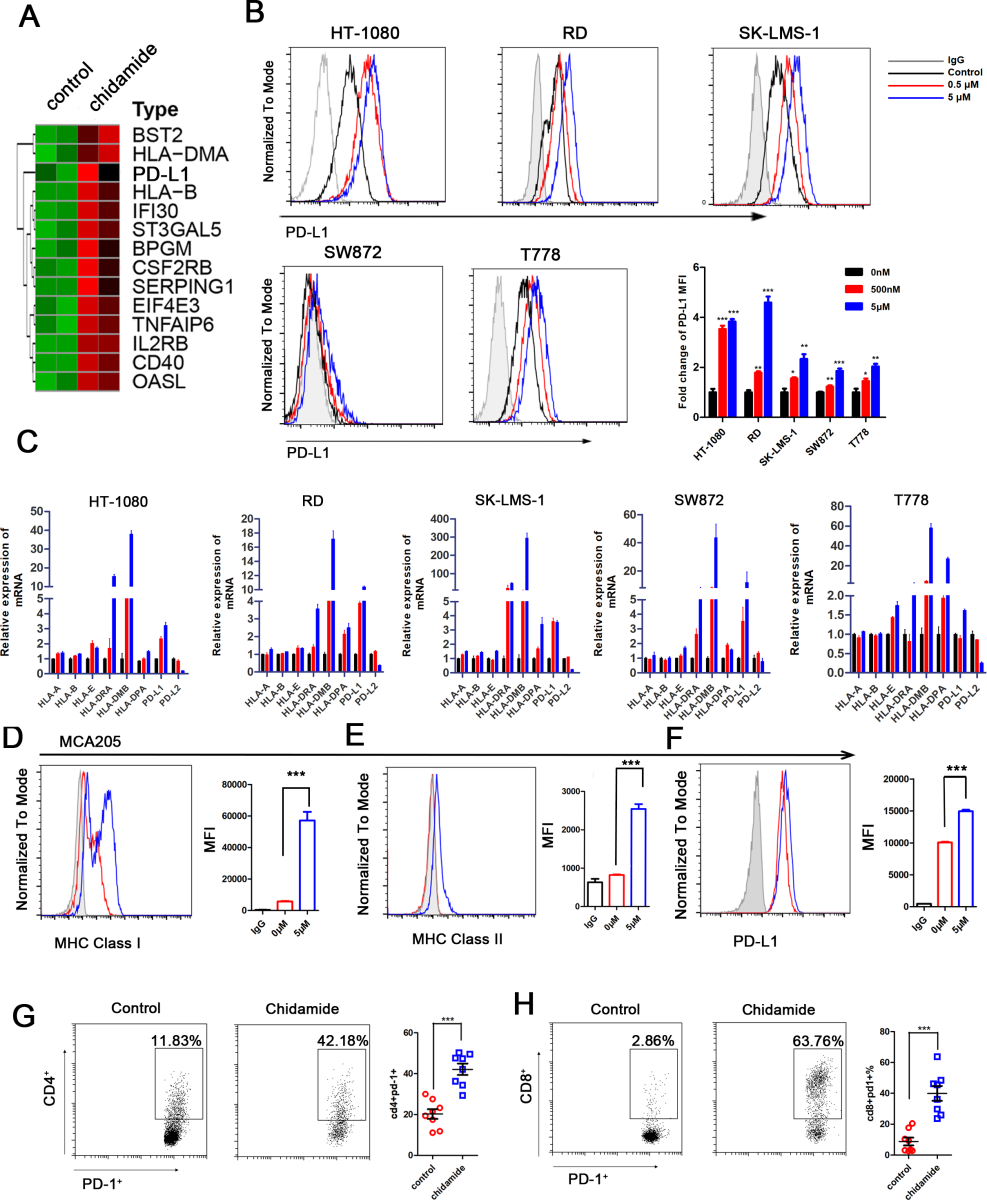
Chidemide induce antigen-presentation and PD-L1 expression in sarcoma. (A) HT-1080 cells were treated with 2.5 µM chidamide in 48 hours, and the global transcriptional genes expression change was measured and analyzed. Heatmaps of significant induced genes by chidamide involved in interferon gamma pathway. (B) HT-1080, RD, SK-LMS-1, SW872 and T778 cells treated with DMSO control and chidamide were subjected to FACS analysis for cell surface PD-L1 expression. Quantification of MFI is shown. Every experiment was run in four independent experiments. ***P<0.001. (C) HT-1080, RD, SK-LMS-1, SW872 and T778 sarcoma cell lines were treated with 500 nM, 5 µM chidamide and DMSO control for 24 hours in vitro, respectively, and HLA-A, HLA-B, HLA-E, HLA-DRA, HLA-DMB, HLA-DPA, PD-L1, and PD-L2 expressions were evaluated. (D–F) MHC class I, MHC class II and PD-L1 expression was analyzed by flow cytometry in MCA205 cells treated with chidamide (0–5 µM) for 48 hours. (G–H) C57BL/6 mice were implanted with 3×10^5^ MCA205 cells subcutaneously and received chidamide or control for 7 days (n=8 per group). MCA205 tumors were isolated to make single cells suspension and subjected to flow cytometry for detection the PD-1 expression in TILs. Representative FACS plots of PD-1 expression in TILs. Quantification of relative CD4^+^PD1^+^ and CD8^+^ PD1^+^ proportion are shown in each group. Three levels of significance (*p<0.05, **p<0.01 and ***p<0.001) were used for all tests. FACS, fluorescence activated cell sorting; MFI, mean fluorescence intensity.

HDAC inhibitors regulate the antitumor immune response in certain malignancies.^28–30^ We observed the upregulation of PD-L1 expression following the knockdown of class I HDACs. Thus, we hypothesized that chidamide would also upregulate PD-L1 expression. To verify this, we used RNA-seq to analyze transcriptional genes expression alteration on chidamide treatment in HT-1080 cell. A hallmark pathway analysis of these upregulated genes revealed 34 significantly affected pathways (online supplemental table S5). Among these pathways we focused on the immune-related pathways. Specifically, 17 genes involved in the allograft rejection pathway were transcriptionally modulated, including HLA-DMA, HLA-DMB, and HLA-DOA, which play important roles in antigen processing and presentation. Importantly, 14 genes involved in the interferon gamma response were upregulated including HLA-B, HLA-C, PD-L1, IL2RB and TNFAIP6, suggesting the reprogramming of the immune microenvironment of sarcoma cells on chidamide inhibition (figure 3A).

PD-L1 expression was evaluated in five sarcoma cell lines with different pathological types using flow cytometry and real-time PCR to confirm the results of this analysis (figure 3B and C). PD-L1 expression was consistently increased in a dose-dependent manner in sarcoma cell lines. In addition to PD-L1, MHC class I and MHC II genes were upregulated, according to the flow cytometry data, indicating that chidamide might enhance antigen presentation to promote antitumor immune response (online supplemental figure S5). Additional HDAC inhibitors including belinostat, panobinostat and vorinostat also showed potency in upregulating MHC class I and class II expression in sarcoma cell lines (online supplemental figure S6). However, PD-L2 expression was not increased by chidamide (figure 3C, (online supplemental figure S6). Next, we detect whether chidamide may exert similar effects on mouse sarcoma tumors. Chidamide also induced MHC class I and class II genes expression and PD-L1 expression in MCA205 cells (figure 3D–3F). Mice bearing xenograft tumors composed of MCA205 cells were treated with chidamide for 7 days (n=8/per group). Then tumors were harvested and subjected to RNA extraction. *pd-l1* mRNA expression and MHC class I and II genes including *H2-Aa*, *H2-Ab*, and *H2-L* mRNA expression were analyzed by q-PCR (online supplemental figure S7). We also harvested the tumor and analyzed tumor-infiltrating lymphocytes (TILs). Both CD8^+^ and CD4^+^ TILs upregulated PD-1 after HDAC inhibition (figure 3G–3H). However, there was no significant changes in CD8^+^ PD-1^+^, and CD4^+^ PD-1^+^ cells were observed in spleen (data not shown). Based on these data, the chidamide treatment enhanced the antigen presentation process and upregulated PD-1/PD-L1 signaling in murine models.

### The combination of chidamide and an anti-PD-1 antibody retards tumor growth and enhances the antitumor response

**Figure 4:**
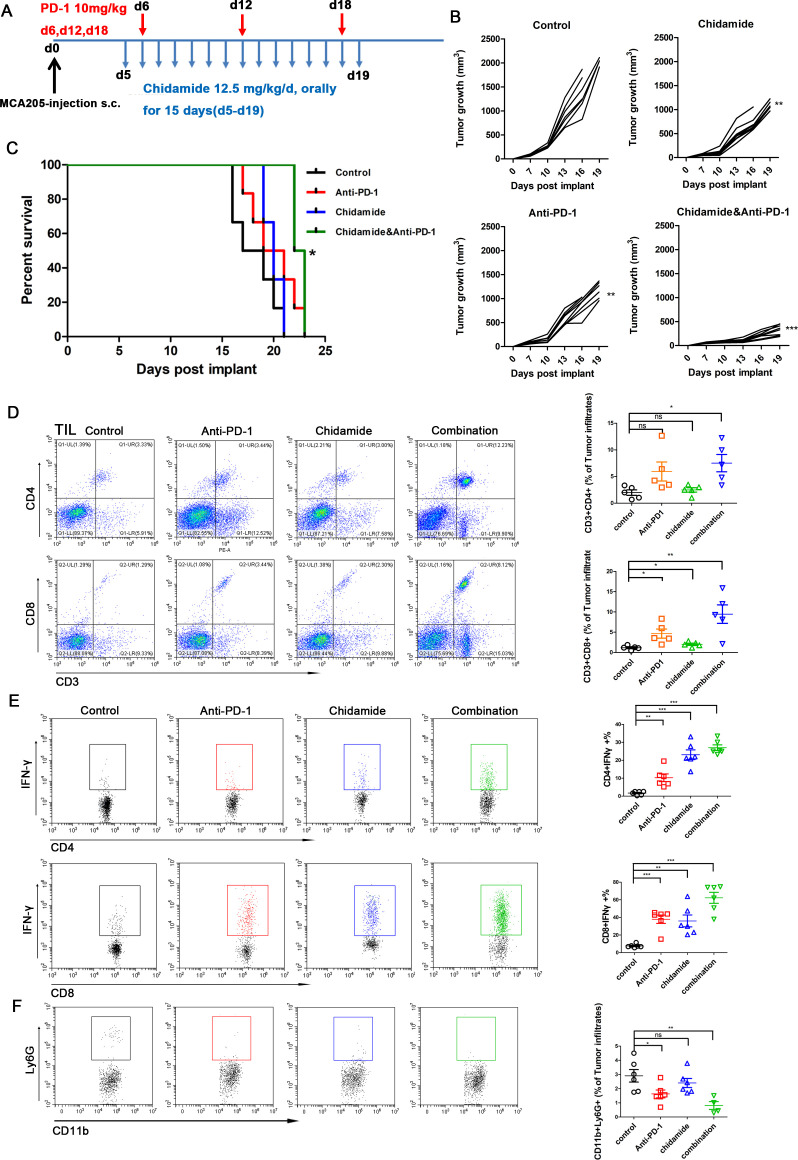
Combining chidamide with PD-1 blockade in vivo results in enhanced survival and leads to increased antitumor activity compared with single therapy and untreated group. C57BL/6 mice were inoculated subcutaneously with MCA205 sarcoma cells. (A) A schematic of treatment for C57BL/6 mice bearing subcutaneous MCA205 tumors. (B) Individual tumor volumes for each group was measured over time (n=8 per group). (C) The survival curve of combination group was significantly different from the control group. Log rank test of survival curve differences were p<0.05. *p<0.05, **p<0.01, and ***p<0.001. (D) After treatment, different group tumors performed FACS to determine CD4 (CD3^+^CD4^+^) and CD8 (CD3^+^CD8^+^) in total viable cells. combination therapy leads to dramatic increase of CD4 (CD3^+^CD4^+^) and CD8 (CD3^+^CD8^+^) percentages of tumor infiltrated cells compared with control group. (E) IFN-γ production in CD4^+^ and CD8^+^ TILs in each group. Combination treatment increased the IFN-γ production in CD4^+^ TIL and CD8^+^ TIL. (F) CD11b^+^Ly6G^+^ percentage cells were attenuated by treated with chidamide and anti-PD-1 most significantly. *P<0.05. **p<0.01. ***p<0.001. TIL, tumor-infiltrating lymphocyte.

We used an immunocompetent murine model of sarcoma cells to determine the therapeutic efficacy of combining the chidamide and an anti-PD-1 antibody. MCA205 cells were subcutaneously injected into C57BL/6 mice. Approximately 4 days later, mice were randomized to treatment with chidamide (12.5 mg/kg), an anti-PD-1 antibody (10 mg/kg), the combination of chidamide and anti-PD-1 antibody or IgG control (n=8 mice /group) as shown in figure 4A. The chidamide group and anti-PD-1 therapy group both exhibited decreased tumor growth compared with the control group (p<0.01). However, the combination group showed a generally significantly (p<0.001) decreased tumor burden compared with that of the IgG control group (figure 4B). Chidamide combined with anti-PD-1 therapy group significantly improved survival (p<0.05) compared with the control group (figure 4C). The control group survived for 18 days, and anti-PD-1 and chidamide-treated groups both survived for 20 days. The combination therapy group survived for 22.5 days. Only the combination group survived significantly longer than the control group (p<0.05).

The percentage of TILs in the four groups was assessed using flow cytometry. The CD3^+^CD8^+^ and CD3^+^CD4^+^ ratios were dramatically increased in the group receiving the combination therapy (figure 4D). The CD3^+^CD8^+^ ratio was also significantly increased in the chidamide group and anti-PD-1 group compared with the control group. However, the percentages of CD4 (CD3^+^CD4^+^) and CD8 (CD3^+^CD8^+^) cells in the spleens of the chidamide or anti-PD-1 groups did not display a significant difference compared with the untreated groups (data not shown). We cultured TILs for 4–6 hours, and then examined IFN-γ production in CD4^+^ and CD8^+^ TILs. The combination treatment significantly increased the production of IFN-γ of TILs compared with monotherapy or the control (figure 4E). Additionaly, the frequency of tumor CD8^+^ and CD4^+^ cells that produce IFN-γ ex vivo in response to MCA205 cells in an ELISPOT assay was significantly increased after exposure to the combination therapy (online supplemental figure S8). Chidamide combined with anti-PD-1 therapy was associated with a significant decrease in the population of MDSCs (CD11b+Ly6G+) compared with the control group (p<0.01). The ratio of MDSCs in the anti-PD-1 therapy group also showed a significant decrease compared with the control group (p<0.05) (figure 4F). Thus, the combination of chidamide with an anti-PD-1 antibody retards tumor growth and enhance the antitumor response by promoting and optimizing the tumor microenvironment.

### Treatment with chidamide and an PD-1 antibody showed a promising antitumor activity in patients with advanced sarcoma presenting HDAC amplification

Based on our preclinical data, we investigated the efficacy and toxicity of chidamide combined with toripalimab (an anti-PD-1 antibody) in patients with advanced STS treated at our institute. These patients were all diagnosed with metastatic disease and at least two lines of therapies had failed.

**Figure 5:**
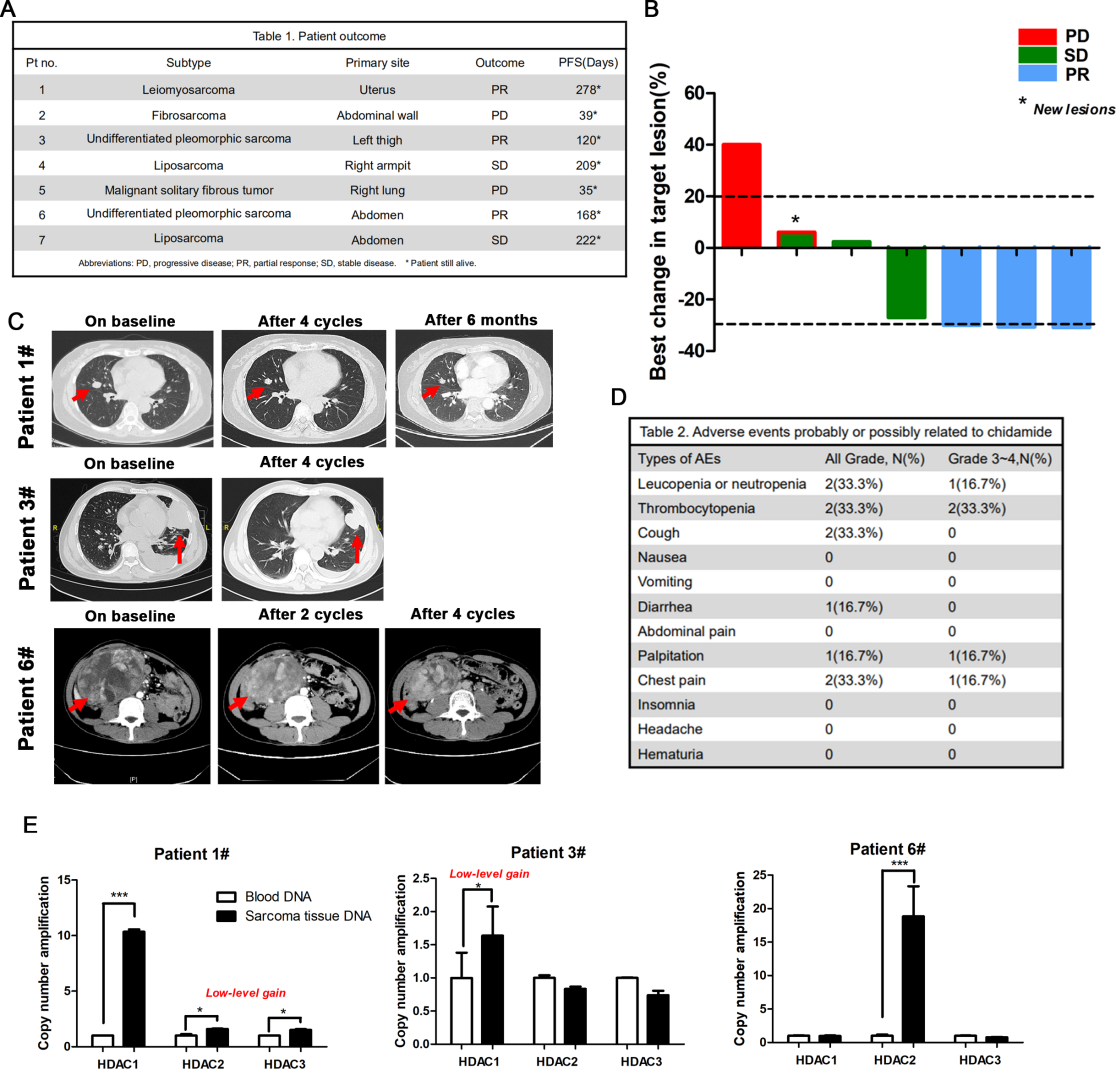
Efficacy and safety of chidamide combined with toripalimab in patients with advanced and metastatic sarcoma. (A) Clinical response and outcome of seven patients after chidamide combined with toripalimab therapy. (B) Best response tumor change in size of target lesions (n=7). Bar length represents increase or decrease in target lesion size. (C) CT of the three patients (patient #1, patient #3 and patient #6 achieved PR). The red arrows denote the target lesions. (D) The summary of safety/toxicity for all treated patients. (E) HDAC1/2/3 genes copy number amplification of sarcoma tissue compared with blood DNA in patient #1, patient #3 and patient #6 who achieved PR. AE, adverse event; HDAC, histone deacetylase; PD, progressive disease, PFS, progression-free survival, PR, partial response; SD, stable disease.

At the time of cut-off, a cohort of seven patients was enrolled to receive chidamide (30 mg/day, twice a week), combined with toripalimab 240 mg, q3wks, and underwent a confirmed response evaluation (figure 5A). The follow-up time for these patients was 40 weeks. Three patients achieved a partial response (PR), two pateints had stable disease, and two patients progressed (figure 5B–C). Currently, all the patients are still alive. All the included patients experienced at least one treatment-related adverse event (AE). The majority of these AEs were mild and tolerable, with no patient discontinuing treatment due to AEs (figure 5D). Then, we performed a q-PCR analysis to detect *HDAC1, HDAC2 and HDAC3* amplification in patients #1, #3 and #6, who achieved PR (figure 5E). In patient #1, the copy numbers of *HDAC2 and HDAC3* displayed a low-level gain, and *HDAC1* was amplified, while in patient #3, the copy numbers of *HDAC1* displayed a low-level gain. In patient #6, *HDAC2* was amplified. However, in patient #2, #4, #5 and #7, the amplification was not detected (online supplemental figure S9). Thus, HDAC class I gene amplification with STS patients who achieved a PR, indicating that chidamide combined with an anti-PD-1 antibody shows a promise as treatment for patients with frequent HDAC amplification in the future.

### Chidamide stimulates PD-L1 expression through the activation of the transcription factor STAT1

**Figure 6:**
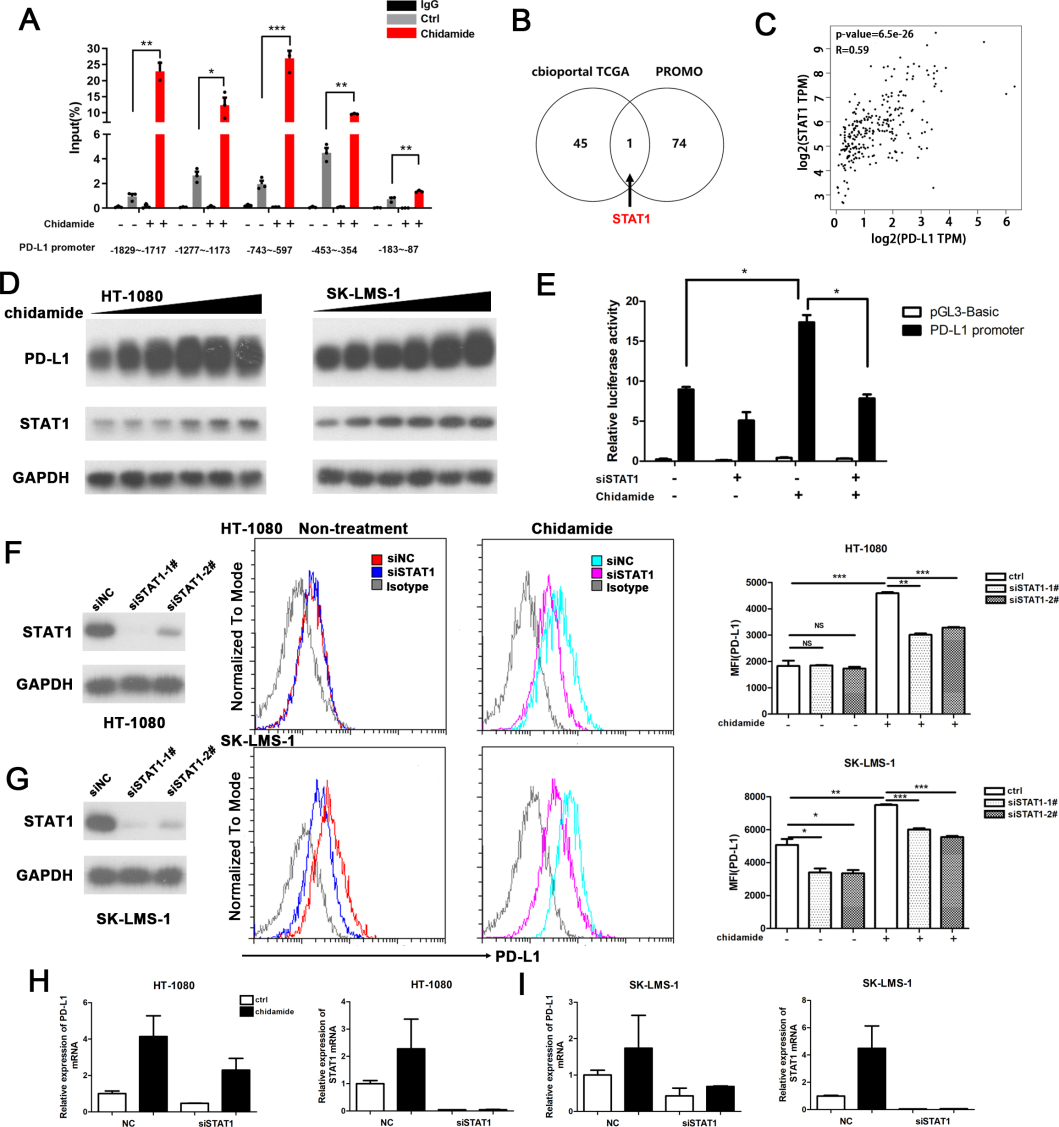
Chidamide stimulate PD-L1 expression through transcriptional factor STAT1 activation. (A) Histone acetylation in PD-L1 promoter. HT-1080 cells were treated with chidamide (5 µM) for 24 hours and subjected to ChIP assay using antiacetylhistone H3K27(Ac-H3K27) antibody followed by q-PCR analysis using primers targeting PD-L1 promoter region. (B) Genes that have coexpression with PD-L1 in soft tissue sarcoma were showed (Spearman expression correlation coefficient of more that 0.5, which were analyzed on the cBioportal website. PROMO bioformatics software was used to predict transcriptional factors that could potentially bind to the PD-L1 promoter. Venn diagram showing that STAT1 was the only candidate gene in both gene sets). (C) Scatter gram showing that mRNA expression correlation PD-L1 and STAT1 from TCGA (SARC, GEPIA website). Spearman correlation coefficients and p values are shown. (D) HT-1080 and SK-LMS-1 cell lines were treated with chidamide in a dose-dependent manner for 24 hours, after that total proteins were prepared to evaluate PD-L1 and STAT1 expression by immunoblotting. (E) HT-1080 cells were transfected with STAT1-targetd or siRNA NC overnight, then they were cotransfected with pGL3-basic vector or the PD-L1 promoter luciferase reporter gene plasmid and the pRL-TK plasmid for 48 hours; chidamide or DMSO was added for another 24 hours. *p<0.05, **p<0.01, ***p<0.001. (F) Left: HT-1080 cells were transfected with siRNA targeting STAT1 for 48 hours; middle: flow cytometry of surface levels of PD-L1 was detected on cells treated or untreated with chidamide for 24 hours; right: quantification of relative MFI of PD-L1. (G) Left: SK-LMS-1 cells were transfected with siRNA targeting STAT1 for 48 hours; middle: flow cytometry of surface levels of PD-L1 was detected on cells treated or untreated with chidamide for 24 hours; right: quantification of relative MFI of PD-L1. Data are mean±SD of three independent experiments. *p<0.05, ***p<0.001 (two-tailed unpaired t-test). (H) mRNA expression of PD-L1 and STAT1 in chidamide treated or untreated HT-1080 cells by transfected NC siRNA or siRNA-STAT1. (I) mRNA expression of PD-L1 and STAT1 in chidamide treated or untreated SK-LMS-1 cells by transfected NC siRNA or siRNA-STAT1. ChIP, chromatin immunoprecipitation; NS, not significant.

We then performed chromatin immunoprecipitation (ChIP) assays to determine the potential chromatin state of the PD-L1 promoter and examined the active histone mark H3K27Ac in sarcoma cells following drug treatment with chidamide for 24 hours. We identified several potential binding sites along the first 2 kbp of the promoter region. Peak acetylation was observed at approximately at −743 to −597 bp upstream of the first exon of the PD-L1 gene (figure 6A).

We wanted to explore the regulatory effect of chidamide on the PD-L1 promotor and hypothesized that chidamide could regulated PD-L1 transcription by affecting the transcription factors that bind to the promoter. Forty-six genes were coexpressed with PD-L1, and genes with correlation coeffcients greater than 0.5 were filtered from the cBioPortal website. On the PROMO website, 75 transcription factors (TF) were predicted to bind to the −2 kb promoter of PD-L1 (online supplemental table S6). Then, a Venn diagram analysis was constructed and showed that STAT1 was the only potential TF (figure 6B). A scatter gram showed that the mRNA expression of PD-L1 and STAT1 was strongly correlated between (SARC, TCGA, Provisional; figure 6C). Western blot analysis also revealed increases in PD-L1 and STAT1 levels following treatment with chidamide (figure 6D). We performed the dual-luciferase reporter assays to explore the regulatory effect of STAT1 on the PD-L1 promoter. As shown in figure 6E, the promoter exhibited significantly enhanced luciferase activity on treatment with chidamide, and this increase was impared by STAT1 silencing. Moreover, knockdown of STAT1 by transfecting siRNAs into chidamide-treated sarcoma cells decreased the surface protein and mRNA expression of PD-L1 (figure 6F, G). Interestingly, knockdown of STAT1 in non-treated HT1080 cells did not significantly change the expression of PD-L1. Base on these data, STAT1 was involved in the chidamide-induced increase in PD-L1 expression.

## Discussion

Recent clinical studies suggest a promising future for immune checkpoint inhibitors, such as an anti-PD-1 antibody, in the treatment of melanoma, but the adoption of this therapy for STS has been slow.^31^ Efforts are underway to determine which STS subtypes will respond to anti-PD-1 antibodies.^32–34^ Tawbi and colleagues reported that undifferentiated pleomorphic sarcoma (UPS) with a higher mutational burden, and relatively high levels of PD-L1 exhibits a better response rate than other types of STS.^9^ Conversely, myxoid/round cell liposarcoma and synovial sarcoma, which both have an immunosuppressive TME, are not responsive to anti-PD-1 therapy.^35^

Immune checkpoint inhibitors may be combined with other agents to enhance the immunogenicity. Multiple studies have reported effects of HDAC inhibitors on augmenting the antitumor immune response of cells. The effects of HDAC inhibitors include increasing the amounts and cytotoxicity of NK and CD8^+^ T cells and modulating of Foxp3^+^ infiltration.^36^ HDAC inhibitors can increase the presentation of tumor-associated antigens and activity of immune-related pathways, resulting in an enhanced antitumor response of T cells.^37^ According to preclinical studies, HDAC inhibitors also upregulate the expression of the immune checkpoints in melanoma.^38^

In the nucleus, histone acetylation and histone deacetylation are in a dynamic balance, and these processes jointly regulate gene expression and silencing. HDACs regulate the silencing of downstream tumor suppressor genes to promote tumor development. However, the amplification of HDAC genes in malignant tumors is not clear. In this project, we reported that the HDAC gene family was frequently amplified in STS by using WES combined with the targeted drug gene screening method.

HDAC class I genes are strongly associated with a poor prognosis. The previous results showed that the expression of class I HDACs, including HDAC1, HDAC2, and HDAC3, is associated with a poor prognosis of patients with sarcoma. Noh *et al* reported a significant association between high expression of HDAC2 and a poor prognosis of patients with hepatocellular carcinoma.^39^ Kawai *et al* found that HDAC1 affects breast cancer progression by promoting cellular proliferation, and it may be a potential target for therapeutic intervention.^40^ We used our own patient cohort as well as samples from TCGA database to show that higher expression of class I type HDACs, including HDAC1, HDAC2 and HDAC3, is associated with worse outcomes in patients with sarcoma.

Based on our findings, the novel HDAC inhibitor chidamide induces the expression of PD-L1 in sarcoma cell lines in vitro and in vivo. We examined whether the tumor-infiltrating CD8^+^ T cells of the combination group were increased, which is effective in boosting antitumor immunity. Treatment with an anti-PD-1 antibody combined with chidamide is associated with significant tumor regression and an improvement in survival time. Moreover, the combination of chidamide and anti-PD-1 therapy led to a greater decrease in MDSCs compared with the other treated and untreated groups. MDSCs are a key immunosuppressive cell population that mediates resistance to immune checkpoint therapies. Our data supported the hypothesis that chidamide decreased the number of MDSCs in the tumor microenvironment of sarcoma. This finding is consistent with previous studies reporting the role of HDAC class I inhibitors.^41 42^

A previous study showed that patients who responded to anti-PD-1 therapy were more likely to have UPS, which has higher densities of infiltrated activated T cells in the tumor microenvironment.^43^ This result has been confirmed in several clinical trials. Burgess *et al* reported the final results of SARC028 expansion cohorts (NCT02301039). They confirmed the clinical activity of pembrolizumab against UPS and dedifferentiated/pleomorphic liposarcoma (LPS) by enrolling additional patients. The ORR of the UPS and LPS cohorts were 23% (9/40) and 10% (4/39), respectively.^44^ In the SARC028 trial, patients who achieved an objective response mainly had UPS or LPS. The median progression-free survival was 18 weeks. However, no patients with leiomyosarcoma achieved an observed objective response.^9^ In the Alliance A091401 study, the median PFS was 1.7 months, and the ORR was 5% for patients who received nivolumab monotherapy, with a response that only occurred in patients with UPS and sarcoma, not otherwise specified.^45^

In our present study, the ORR of all the patients was 42.9% after treatment with anti-PD-1 combined with chidamide. The median PFS had not been reached at the time of analysis. No patients had died. Three patients achieved a PR—two of whom had UPS and the other was diagnosed with leiomyosarcoma. The subtype of two patients who achieved SD was liposarcoma. Therefore, chidamide combined with an anti-PD-1 antibody showed efficacy in treating patients with pathological subtypes other than UPS and patients with frequent HDAC amplification.

However, our study has some limitations. First, because sarcomas are a heterogeneous group of mesenchymal tumors with more than 50 subtypes, the efficacy of chidamide and anti-PD-1 therapy in all types of sarcoma cells and animal models is difficult to examine. Thus, efforts to confirm the activity of chidamide with anti-PD-1 therapy in clinical trials are ongoing. Then, an assessment of the subtypes of sarcoma that are sensitive to PD-1 antibodies remain an important research direction.

In summary, this study investigated the HDAC inhibitor chidamide combined with an immune checkpoint inhibitor in patients with STS, which has not been reported previously. We reported that the HDAC gene family was frequently amplified in STS by using WES combined with the targeted drug gene screening method. The results demonstrated that chidamide may alter the expression of PD-L1 in malignant cells, and it also change T cell subpopulations in the tumor microenvironment. Chidamide, which increase PD-L1 expression, enhance the therapeutic efficacy in patients with sarcoma. Taken together, since multiple HDAC inhibitors and anti-PD-1 agents are now approved, combination therapy with these two agents would represent a new approach with considerable potential to treat STS. Based on our results, this combination therapy represents a potential strategy for increasing the response of patients with STS to immunotherapy.
