# Supplementary material for: Frequent amplification of HDAC genes and efficacy of HDAC inhibitor chidamide and PD-1 blockade combination in soft tissue sarcoma
Source: J Immunother Cancer. 2021 Feb 26;9(2):e001696. doi: 10.1136/jitc-2020-001696 (PMC7919591; doi:10.1136/jitc-2020-001696)
Supplement: Supplementary data [file jitc-2020-001696supp001.pdf]

Supplementary materials for

Frequent amplification of HDAC genes and efficacy of HDAC inhibitor Chidamide and PD-1 blockade combination in soft tissue sarcoma

Yi Que[1,2#], Xiaolong Zhang[3#], Zexian Liu[3], Jingjing Zhao[1], Qiuzhong Pan[1], Xizhi Wen[1], Wei Xiao[4], Bushu Xu[1], Dongchun Hong[1], Tianhui Guo[1], Lujun Shen[5], Weijun Fan[5], Huoying Chen[6], Desheng Weng[1], Hairong Xu[7], Penghui Zhou[3], Yizhuo Zhang[2*], Xiaohui Niu[7*], Xing Zhang[1*]

[1] Department of Medical Melanoma and Sarcoma, State Key Laboratory of Oncology in South China, Collaborative Innovation Center for Cancer Medicine, Sun Yat-sen University Cancer Center, Guangzhou, China.
[2] Department of Pediatric Oncology, State Key Laboratory of Oncology in South China, Collaborative Innovation Center for Cancer Medicine, Sun Yat-sen University Cancer Center, Guangzhou, China.
[3] State Key Laboratory of Oncology in South China, Collaborative Innovation Center for Cancer Medicine, Sun Yat-sen University Cancer Center, Guangzhou, China.
[4] Department of Hematological Oncology, State Key Laboratory of Oncology in South China, Collaborative Innovation Center for Cancer Medicine, Sun Yat-sen University Cancer Center, Guangzhou, China.
[5] Department of Minimally Invasive Interventional Therapy, State Key Laboratory of Oncology in South China, Collaborative Innovation Center for Cancer Medicine, Sun Yat-sen University Cancer Center, Guangzhou, China.
[6] Department of Laboratory Medicine, The Second Affiliated Hospital of Guilin Medical University, Guilin, China.
[7] Department of Orthopedic Oncology Surgery, Beijing Ji Shui Tan Hospital, Peking University, Beijing, China.

This PDF file includes: Supplementary Figure 1, Supplementary Figure 2, Supplementary Figure 3, Supplementary Figure 4, Supplementary Figure 5, Supplementary Figure 6, Supplementary Figure 7, Supplementary Figure 8, Supplementary Figure 9, Supplementary Table 1, Supplementary Table 2, Supplementary Table 3, Supplementary Table 4, Supplementary Table 5, Supplementary Table 6, and Supplementary Methods.

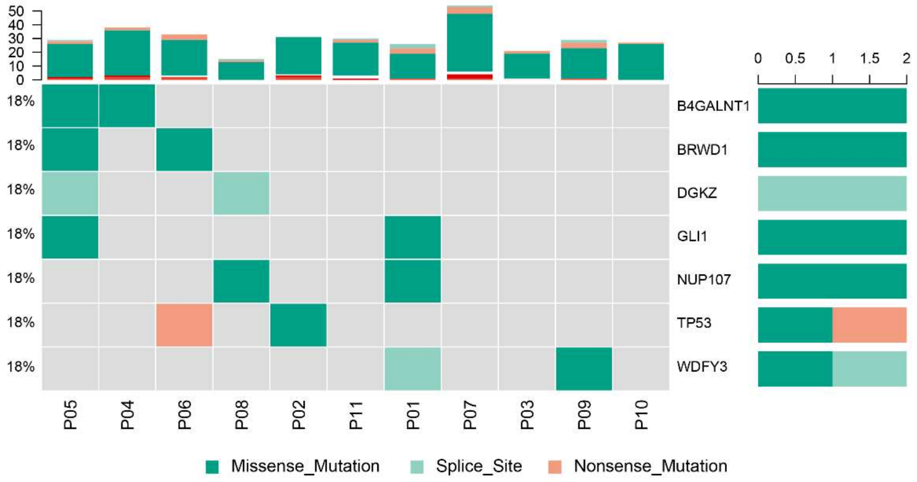

Figure. S1 Oncoplot depicting shared mutated genes (mutated in more than 1 sample) sorted and ordered by decreasing frequency.

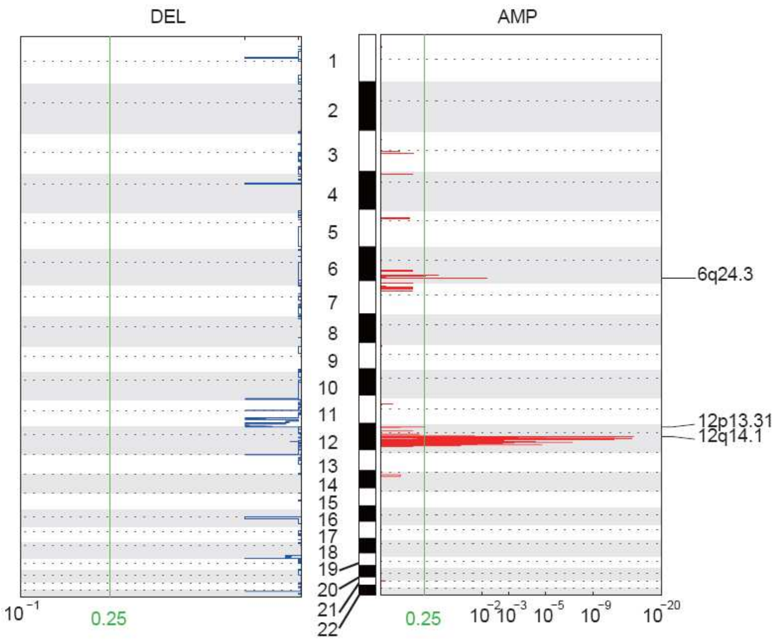

Figure. S2 Focal amplification/deletion patterns of somatic copy-number alterations. The amplification peaks are shown in red and deletion peaks are shown in blue. The significant threshold (FDR<0.25) were shown as vertical green lines.

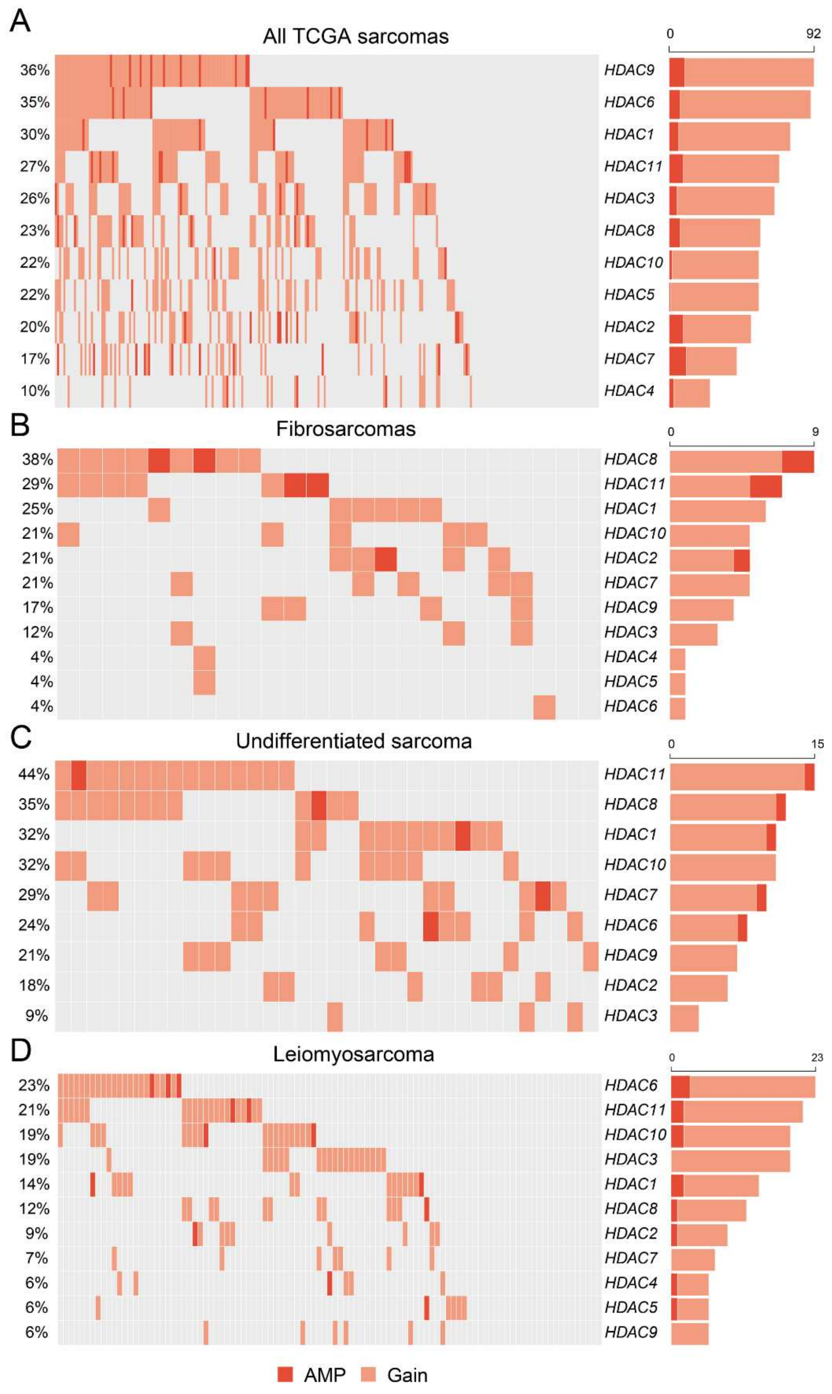

Figure S3. The HDAC gene family was extensively amplified in TCGA sarcoma samples. A) The HDAC gene family was amplified or gained in 197(76.65%) patients in 257 sarcoma patients in TCGA database. B) In fibrosarcoma samples, the HDAC

gene family was amplified or gained in 22 of 24 samples (91.67%). C) In undifferentiated_sarcoma samples, the HDAC gene family was amplified or gained in 34 of 34 samples (100%). D) In leiomyosarcoma samples, the HDAC gene family was amplified or gained in 76 of 101 samples (75.25%).

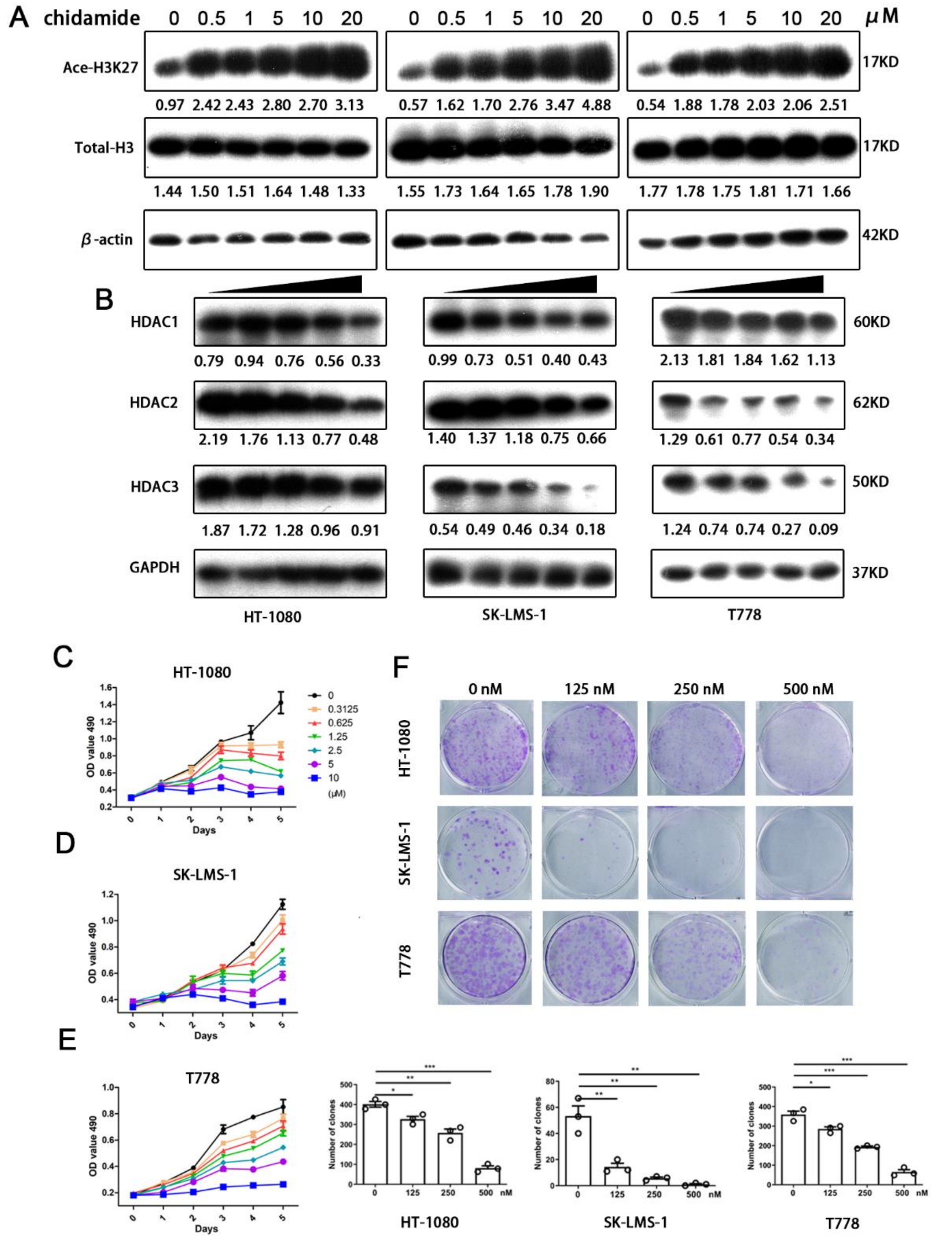

Figure S4. Chidamide exerts potential inhibitory effects on class I HDACs

A) HT-1080, SK-LMS-1, T778 sarcoma cells were cultured for 24 hours in the presence of indicated chidamide (0, 0.5, 1, 5, 10, 20μM). Cells were washed, lysed and analyzed by immunoblotting for acetylated-histone H3K27, total histone 3 and β-actin). B) HT-

1080, SK-LMS-1, T778 sarcoma cells were cultured for 48 hours in the presence of indicated chidamide (0, 0.5, 1, 5, 10μM). Cells were washed, lysed and analyzed by immunoblotting for HDAC1, HDAC2, HDAC3 and GAPDH. C-E) HT-1080, SK-LMS-1 and T778 cells were treated at different dose at 0, 1, 2, 3, 4, 5 day, then MTT assays were performed and absorbance was read at 490 nm. F) Representative images of colony forming capacity of sarcoma cell lines (HT-1080, SK-LMS-1, T778) following chidamide treatment (0-500 nM) at 14 days using crystal violet staining.

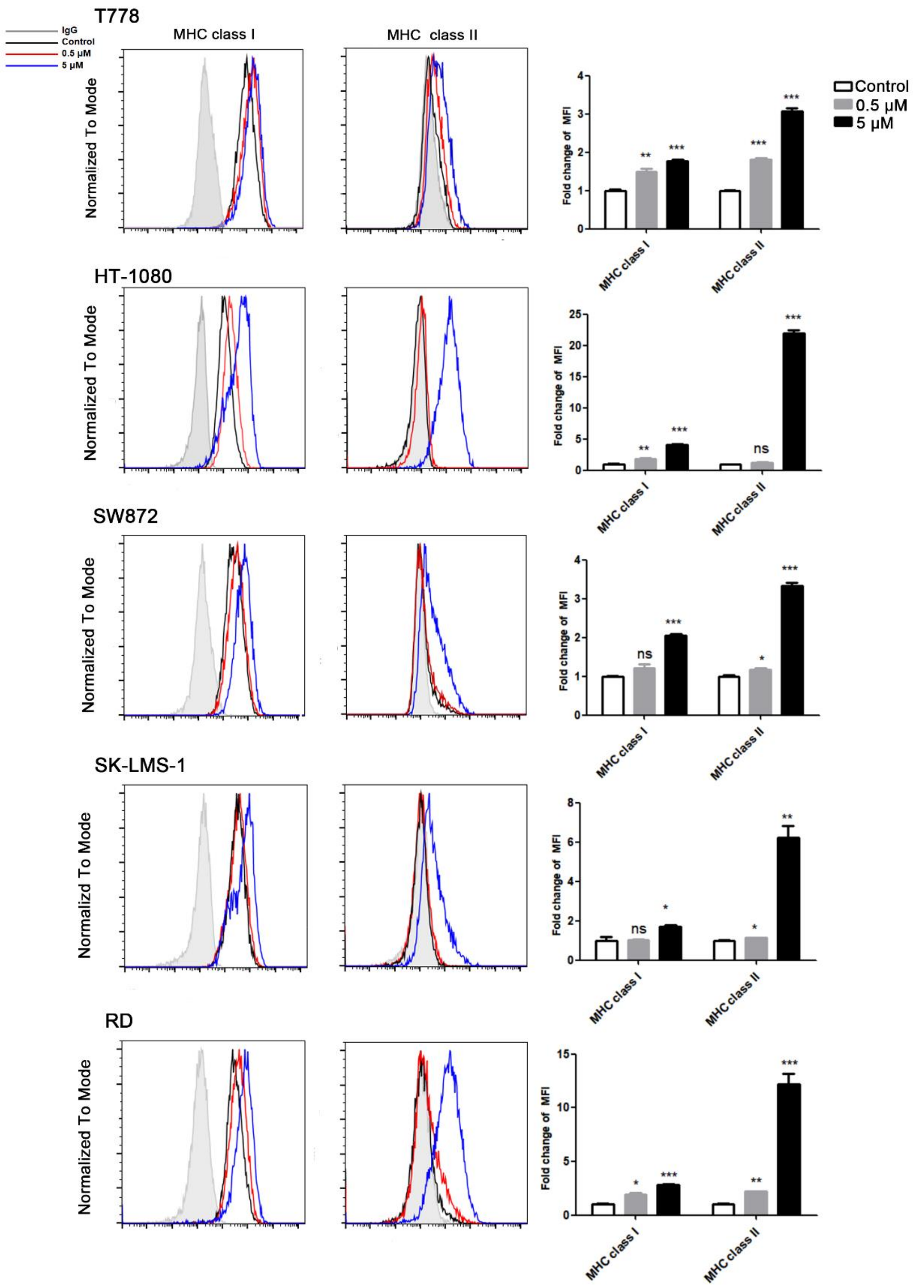

Figure S5. Chidamide upregulates MHC class I and II levels in human sarcoma cell lines. T778, HT-1080, SW872, SK-LMS-1 and RD cells treated with DMSO control and chidamide were subjected to FAC analysis for cell surface MHC class I and

II expression. Quantification of MFI is shown.

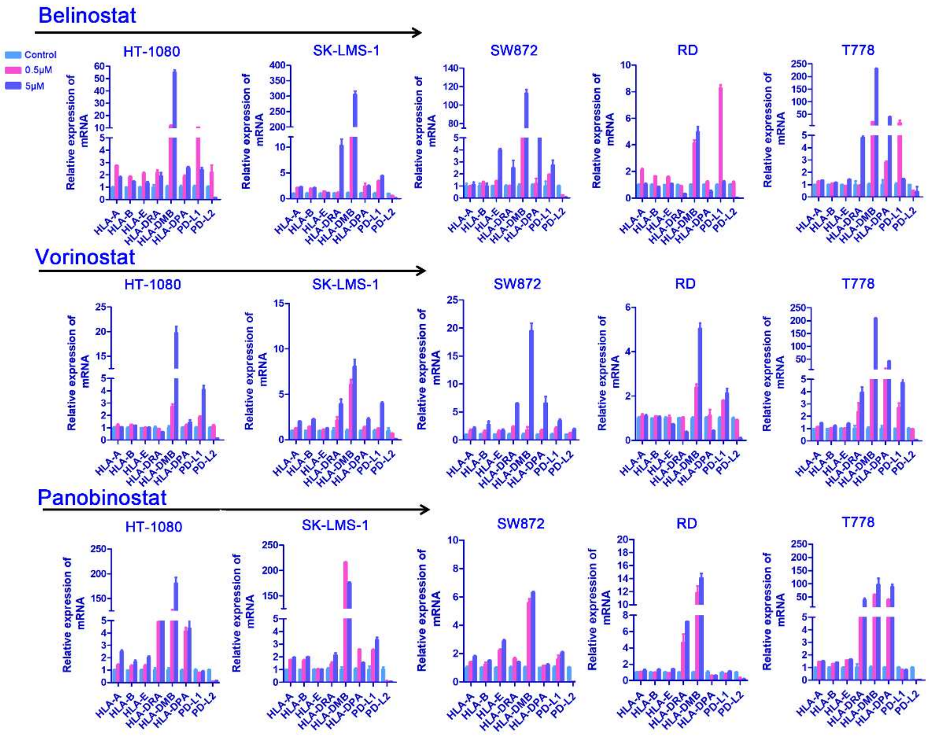

Figure S6. Belinostat, vorinostat and panobinostat upregulate MHC class I and II levels in human sarcoma cell lines. HT-1080, RD, SK-LMS-1, SW872 and T778 sarcoma cell lines were treated with 500 nM, 5 μM HDAC inhibitors (including belinostat, vorinostat and panobinostat) and DMSO control for 24 hours *in vitro* respectively and mRNA expression of HLA-A, HLA-B, HLA-E, HLA-DRA, HLA-DMB, HLA-DPA, PD-L1, PD-L2 was evaluated.

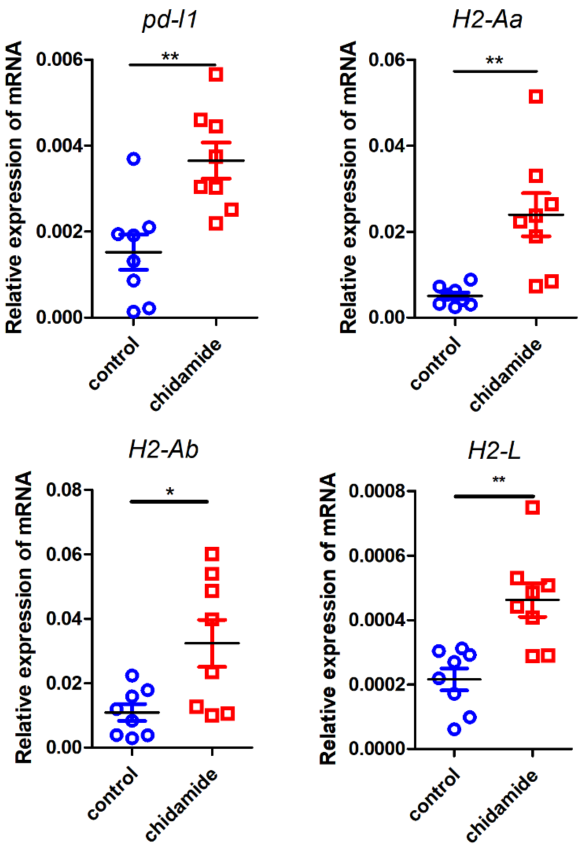

Figure S7. Chidamide upregulates MHC class I and II gene levels of sarcoma *in vivo*. C57BL/6 mice were implanted with 3×10^5 MCA205 cells subcutaneously and received chidamide or control for 7 days (n=8/ per group). Then tumors were harvested and subjected to RNA extraction. *pd-l1* mRNA expression and MHC class I &II genes including *H2-Aa*, *H2-Ab*, *H2-L* mRNA expression was analyzed by qPCR.

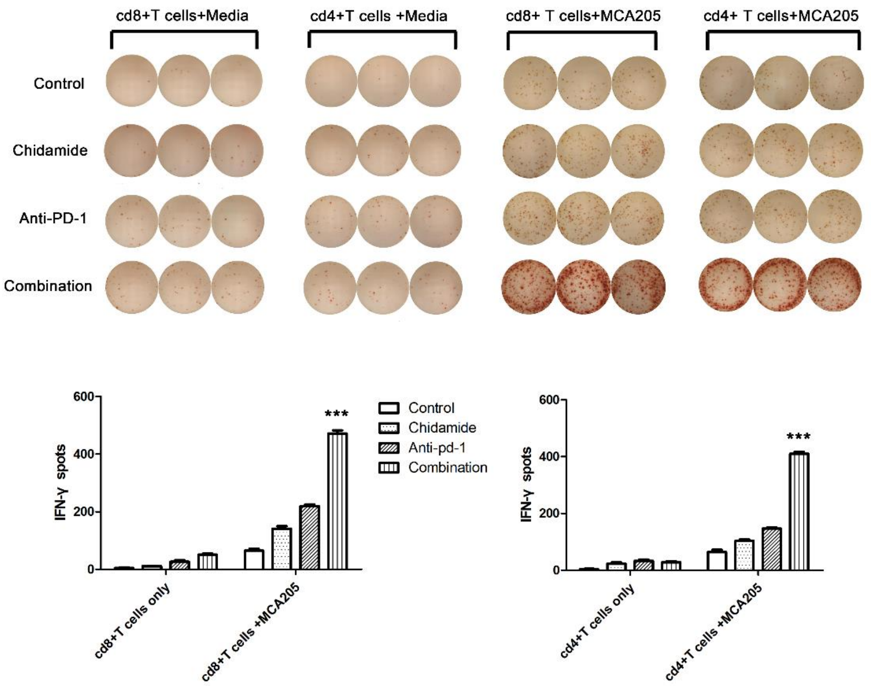

Figure S8. Chidamide combined with anti-PD-1 enhances the function of tumor-infiltrating T cells. IFNγ ELISPOT of tumor CD8+ and CD4+ T cells cultured alone, with or without MCA205 as indicated. T cells, per group, were pooled and samples were run in triplicate (mean± SE). *** P<0.001

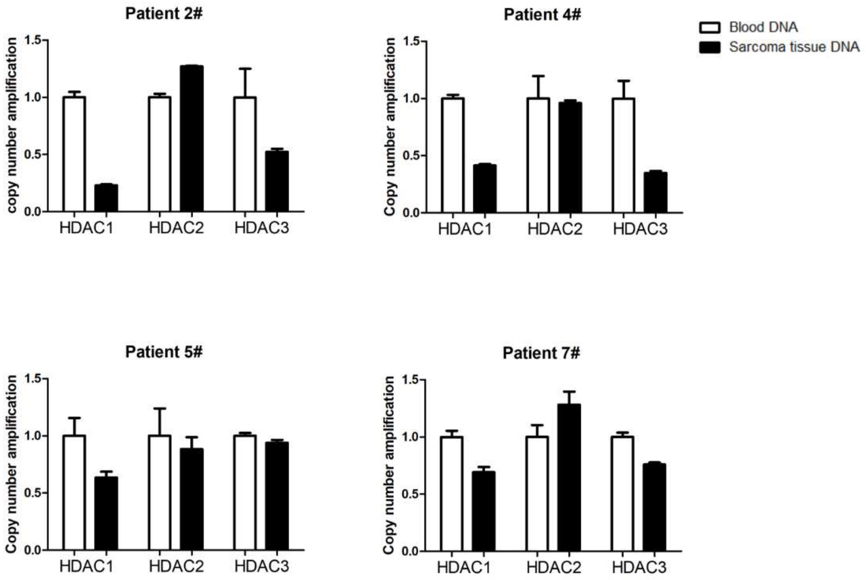

Figure S9. The status of HDAC1/2/3 genes copy number amplifications in sarcoma tissue compared with blood DNA in patient 2#, 4#,5# and 7#

Table S1 Characteristics of 49 patients with sarcoma who have been evaluated the expression and prognosis of class I HDACs

**Characteristics**	N(%)
**Gender**	
Male	33(67.3%)
Female	16(32.7%)
**Age**	
<60	29(59.2%)
≥60	20(40.8%)
**Pathology**	
Liposarcoma	10(20.4%)
Fibrosarcoma	10(20.4%)
Synovial sarcoma	4(8.2%)
Leiomyosarcoma	2(4.1%)
UPS	11(22.4%)
MPNST	5(10.2%)
Others	7(14.3%)
**Site**	
Trunk	16(32.7%)
Head and neck	7(14.3%)
Extremity	14(28.6%)
Intra-abdominal	12(24.5%)
**Depth**	
Deep	40(81.6%)
Superficial	9(18.4%)
**Size**	
<5cm	18(36.7%)
≥5cm	31(63.3%)
**Grade**	
G1	2(4.1%)
G2	16(32.7%)
G3	31(63.3%)
**Stage**	
IA~IB	2(4.1%)
IIA~IIB	24(49.0%)
III~IV	23(47.0%)

Table S2 Sequences of primers used for quantitative real-time PCR

Gene	Forward primer (5'-3')	Reverse primer (5'-3')
HLA-A	GATTACATCGCCTTGAACGAGG	AGAGACAGCGTGGTGAGTCAT
HLA-B	CAGTTCGTGAGGTTCGACAG	CAGCCGTACATGCTCTGGA
HLA-E	TTCCGAGTGAATCTGCGGAC	GTCGTAGGCGAACTGTTCATAC
PD-L1	GGACAAGCAGTGACCATCAAG	CCCAGAATTACCAAGTGAGTCCT
PD-L2	ATTGCAGCTTCACCAGATAGC	AAAGTTGCATTCCAGGGTCAC
HLA-DRA	TCTGGCGGCTTGAAGAATTTG	GGTGATCGGAGTATAGTTGGAGC
HLA-DMB	ACCTGTCTGTTGGATGATGCT	CGCAAGGGGCCATCTTATTCT
HLA-DPA	ATGCGCCCTGAAGACAGAATG	ACACATGGTCCGCCTTGATG
ACTB	GTGAAGGTGACAGCAGTCGGT	AAGTGGGGTGGCTTTTAGGA
pd-l1	GCTCCAAAGGACTTGTACGTG	TGATCTGAAGGGCAGCATTTC
H2-Aa	TCAGTCGCAGACGGTGTTTAT	GGGGGCTGGAATCTCAGGT
H2-Ab	AGCCCCATCACTGTGGAGT	GATGCCGCTCAACATCTTGC
H2-L	GATGCAGAGCATTACAGGGC	GCCAGGTCAGGGCAATGTC
actb	GGCTGTATTCCCCTCCATCG	CCAGTTGGTAACAATGCCATGT

Table S3 Sequences of primers used for analysis of HDAC amplification

Gene	Forward primer (5’-3’)	Reverse primer (5’-3’)
HDAC1	CCACCCATTCTTCCCGTTCT	AAGCAGGCACTTGGCATTTC
HDAC2	GAGGTGGCTACACAATCCGT	GGGAATCTCACAATCAAGGGC
HDAC3	CTCTCCCAAGGCCTGACAAT	GGGCCCACTGCCAATAATGT
GAPDH	CCCCCGGTTTCTATAAATTGAGC	AAGAAGATGCGGCTGACTGT

Table S4 ChIP primers used for the analysis of the PD-L1 promoter

ChIP Site	Forward primer (5'-3')	Reverse primer (5'-3')
-1829~-1717	CGCAAATCACTGAGCAGCAA	GCAGTGTTCAGGGTCTACCT
-1277~-1173	CCCAGCTCAGATGTTCCTTCT	TGTCATAACCAATGCAAGGGC
-743~-597	TTGGGCCCATTCACTAACCC	AAGAACTTCCCATCCCGAGC
-453~-354	TGGGTCTGCTGCTGACTTTT	AGGCGTCCCCCTTTCTGATA
-183~-87	ACTGGACTGACATGTTTCACTT	ATCGGCGGAAGCTTTCAGTT

Table S5 Hallmark pathway analysis of upregulated genes by the transcriptome revealed 34 significantly affected pathways after chidamide treatment

GENE SETS	GENES	P-VALUE
HALLMARK_EPITHELIAL_MESENCHYMAL_TRANSITION	41	5.77E-20
HALLMARK_ESTROGEN_RESPONSE_EARLY	32	9.40E-13
HALLMARK_KRAS_SIGNALING_DN	31	5.00E-12
HALLMARK_IL2_STAT5_SIGNALING	30	2.56E-11
HALLMARK_APICAL_JUNCTION	29	1.26E-10
HALLMARK_ESTROGEN_RESPONSE_LATE	29	1.26E-10
HALLMARK_INFLAMMATORY_RESPONSE	27	2.70E-09
HALLMARK_HYPOXIA	26	1.18E-08
HALLMARK_MYOGENESIS	26	1.18E-08
HALLMARK_UV_RESPONSE_UP	22	4.43E-08
HALLMARK_COAGULATION	20	9.16E-08
HALLMARK_COMPLEMENT	24	1.96E-07
HALLMARK_KRAS_SIGNALING_UP	24	1.96E-07
HALLMARK_XENOBIOTIC_METABOLISM	23	7.46E-07
HALLMARK_UV_RESPONSE_DN	19	8.40E-07
HALLMARK_APOPTOSIS	18	1.70E-05
HALLMARK_TNFA_SIGNALING_VIA_NFKB	19	9.62E-05
HALLMARK_TGF_BETA_SIGNALING	9	1.06E-04
HALLMARK_WNT_BETA_CATENIN_SIGNALING	7	6.16E-04
HALLMARK_ALLOGRAFT_REJECTION	17	7.93E-04
HALLMARK_HEDGEHOG_SIGNALING	6	1.51E-03
HALLMARK_GLYCOLYSIS	16	2.09E-03
HALLMARK_HEME_METABOLISM	16	2.09E-03
HALLMARK_APICAL_SURFACE	6	4.30E-03
HALLMARK_FATTY_ACID_METABOLISM	12	1.06E-02
HALLMARK_PEROXISOME	9	1.14E-02
HALLMARK_IL6_JAK_STAT3_SIGNALING	8	1.18E-02
HALLMARK_INTERFERON_GAMMA_RESPONSE	14	1.20E-02
HALLMARK_P53_PATHWAY	14	1.20E-02
HALLMARK_PANCREAS_BETA_CELLS	5	1.29E-02
HALLMARK_BILE_ACID_METABOLISM	9	1.79E-02
HALLMARK_ANDROGEN_RESPONSE	8	2.54E-02
HALLMARK_NOTCH_SIGNALING	4	2.54E-02
HALLMARK_ADIPOGENESIS	13	2.60E-02

Table S6 The genes predicted in Figure 6B

**Input sequence**
CATCTGTTTTGCTTTACATATTTTTCTGAGGTAATAAAATTTCTCTTTTTCTAAACACA GCCTGTTTTTCAATCTCCGGGTAGTTGATCAATTGTATGGGAAAATGAATGGCTGAA GGGTAGAAACAGGTGGGAAAGATGAACAAAAACACGAATCCTCACATTACTAATAC GCAAATCACTGAGCAGCAAGCTGAGCAAATACCCTCAATTCCCATCAGCAACTTTAG AGAAAGGCAAATTCCGTTTGCCTCATTGATCATTTAGGTAGACCCTGAACACTGCTT TCATAAAACAAAAACAAAATACCCATCCCCAGTTTAAAAAATTATTCATAGATCATCC AGGCCATCTAGGAGGATATGATTTAATCCTGGCTACTTGGTAAATTATTTGCCCAAGT TAACTCAGCTAGTTAGTGGTAGATGGCTCTGAAGCCAGTTGTTTTTTTTTTGTTTTGTT TTTTGCAGACCTCAAGAGTCATGATGAACTAGCAGATCATAAAGTTTATGCCCTGGG TCTTGACCATTTTTAGAAAAATAAAACATTAAATGAAAATATCAGAGGGCATTGCAG ATAGTAGATCTAAGTATTTTTTCATGAAACTTGTTGTACATGTGTGTGTCATACACAG ACTATATATATGCAGTACCTGTAAACTGTATTGCCACATAATGTCTATATTTTCCTAGAG GTCACAGTCACCAAAGTTGGGAAGTCACCCAACTTCGGGAACTTTGGGAAGTCACC CAAACTTACAGTCACCAAAATTGCTCTATTCTACTATGTGACCTCAAAAGTGATTTGA AAGAAGGAACATCTGAGCTGGGCCCAAACCCTATTGCAATTTTATTGGGGCCAAAG AGAACTCCATGCTCCTGCCAAATCAAGGCAGTGTCAGCCTCAATAATTTCCCAGATA AAAATAAAAATCTGTGATACAATCAGAATGTGAAAATTCTTATTTTGGAAGCAAATG TCATAACCAATGCAAGGGCTATCTCAATATTCATTCATTATGCAGTA
**Factors predicted by PROMO in this sequence**
**NAME; MATRIX**
C/EBPbeta[T00581];GR-alpha[T00337];GR-beta[T01920];TFIID [T00820]; HNF-3alpha [T02512]; HOXD9 [T01424];HOXD10 [T01425]; FOXP3 [T04280]; PR B [T00696]; PR A[T01661];TFII-I [T00824]; C/EBPalpha [T00105]; IRF-1 [T00423]; NF-AT1[T00550]; XBP-1 [T00902]; ENKTF-1 [T00255]; VDR[T00885]; PXR-1:RXR-alpha [T05671]; GR [T05076]; RXR-alpha [T01345]; STAT4 [T01577]; c-Ets-1 [T00112]; YY1 [T00915]; RBP-Jkappa [T01616]; c-Myb [T00137]; AP-2alphaA [T00035]; MEF-2A [T01005]; PEA3 [T00685]; NF-1 [T00539]; Pax-5 [T00070]; p53 [T00671]; PPAR-alpha:RXR-alpha [T05221]; NFI/CTF [T00094]; HNF-1A [T00368]; HNF-1B [T01950]; AP-1 [T00029]; c-Jun [T00133]; c-Fos [T00123]; ER-alpha [T00261]; GATA-1 [T00306]; NF-AT2 [T01945]; STAT1beta [T01573]; COUP-TF1 [T00149]; T3R-beta1 [T00851]; SRY [T00997]; TCF-4E [T02878]; HNF-4alpha [T03828]; LEF-1 [T02905]; ATF3 [T01313]; IRF-2 [T01491]; c-Ets-2 [T00113]; RAR-beta:RXR-alpha [T05420]; RAR-beta [T00721]; NF-Y [T00150]; RelA [T00594]; E2F [T00221]; GCF [T00320]; HNF-1C [T01951]; E2F-1 [T01542]; Elk-1 [T00250]; TCF-4 [T02918]; ELF-1 [T01113]; STAT5A [T04683]; POU2F1 [T00641]; Sp1 [T00759]; AR [T00040]; GATA-2 [T00308]; TBP [T00794]; PU.1 [T02068]; NF-kappaB [T00590]; EBF [T05427]; GATA-3 [T00311]; USF2 [T00878]; Ik-1 [T02702]; POU2F2 (Oct-2.1) [T00646];

Supplementary methods

DNA extraction and next generation sequencing

Hematoxylin and eosin stained slides were reviewed by experienced sarcoma pathologists to determine the subtype of each sample. DNA was extracted from thick serial sections cut from fresh-frozen tumor tissue samples as well as matched blood samples using a DNeasy Kit (QIAGEN) according to the manufacturer's protocol. DNA was quantified by Qubit (Life Technologies), and DNA integrity was examined by agarose gel electrophoresis. Paired-end DNA libraries were prepared according to the manufacturer's instructions (Agilent). The adapter-modified gDNA fragments were enriched by six PCR cycles. Whole exome capture was carried out using a SureSelect Human All Exon V6 Kit (Agilent). After DNA quality assessment, the captured DNA library was sequenced on an Illumina Hiseq Xten sequencing platform (Illumina) according to the manufacturer's protocol for paired-end 150 bp reads (WuXi AppTec, Shanghai), achieving an average coverage of 200×.

Data processing

Paired-end clean reads in the FastQ format generated by the Illumina pipeline were aligned to the reference human genome (UCSC hg19) by the Burrows-Wheeler Aligner (BWA)[1] to obtain the original mapping results stored in BAM format. SAMtools[2], Picard (http://broadinstitute.github.io/picard/), and GATK[3] were used to sort BAM files and perform duplicate marking, local realignment, and base quality recalibration to generate final BAM files for the computation of the sequence coverage and depth. The somatic SNVs and InDels were detected by MuTect2[4]. Variant position, type and presentation in known databases of detected SNVs, such as dbSNP and the 1000 Genomes Project (1000GP)[4], were annotated using ANNOVAR[5]. Variants with frequencies greater than 0.1% in the 1000GP, the Exome Aggregation Consortium (ExAC)[6] and the Exome Sequencing Project (ESP; esp6500siv2) were ignored in

further analyses. EXCAVATOR2[7] was utilized to detect somatic CNVs. Somatic copy number (segmented copy number measured by an Affymetrix Genome-Wide Human SNP Array 6.0) data for TCGA liposarcoma (57 patients) was obtained from UCSC Xena (http://xena.ucsc.edu). GISTIC2[8] analysis was used to identify recurrent amplification and deletion peaks in both our own data and the TCGA data. We defined "amplification (AMP)", "gain", "loss" and "deletion (DEL)" at the gene level based on the "all_thresholded.by_genes.txt" file provided by GISTIC2 as follows:

- -2 indicates a deep deletion (DEL), possibly a homozygous deletion;
- -1 indicates a shallow loss, possibly a heterozygous deletion;
- 0 is diploid;
- 1 indicates a low-level gain (a few additional copies, often broad);
- 2 indicate a high-level amplification (AMP; more copies, often focal).

The drug-targeted gene set was selected from distinct public databases, including the National Cancer Institute (NCI)-Match (http://www.cancer.gov/about-cancer/treatment/clinical-trials/nci-supported/nci-match) and MD Anderson Personalized Cancer Therapy (PCT) (https://pct.mdanderson.org) criteria, as well as FDA-approved kinase inhibitors from DSigDB[9].

1. Li H, Durbin R: **Fast and accurate short read alignment with Burrows-Wheeler transform**. *Bioinformatics* 2009, **25**(14):1754-1760.
2. Li H, Handsaker B, Wysoker A, Fennell T, Ruan J, Homer N, Marth G, Abecasis G, Durbin R, Genome Project Data Processing S: **The Sequence Alignment/Map format and SAMtools**. *Bioinformatics* 2009, **25**(16):2078-2079.
3. McKenna A, Hanna M, Banks E, Sivachenko A, Cibulskis K, Kernytsky A, Garimella K, Altshuler D, Gabriel S, Daly M *et al*: **The Genome Analysis Toolkit: a MapReduce framework for analyzing next-generation DNA sequencing data**. *Genome research* 2010, **20**(9):1297-1303.
4. Cibulskis K, Lawrence MS, Carter SL, Sivachenko A, Jaffe D, Sougnez C, Gabriel S, Meyerson M, Lander ES, Getz G: **Sensitive detection of somatic point mutations in impure and heterogeneous cancer samples**. *Nature biotechnology* 2013, **31**(3):213-219.
5. Wang K, Li M, Hakonarson H: **ANNOVAR: functional annotation of genetic variants from high-throughput sequencing data**. *Nucleic acids research* 2010, **38**(16):e164.
6. Lek M, Karczewski KJ, Minikel EV, Samocha KE, Banks E, Fennell T, O'Donnell-Luria AH, Ware JS, Hill AJ, Cummings BB *et al*: **Analysis of protein-coding genetic variation in 60,706 humans**. *Nature* 2016, **536**(7616):285-291.

7. D'Aurizio R, Pippucci T, Tattini L, Giusti B, Pellegrini M, Magi A: **Enhanced copy number variants detection from whole-exome sequencing data using EXCAVATOR2**. *Nucleic acids research* 2016, **44**(20):e154.
8. Mermel CH, Schumacher SE, Hill B, Meyerson ML, Beroukhim R, Getz G: **GISTIC2.0 facilitates sensitive and confident localization of the targets of focal somatic copy-number alteration in human cancers**. *Genome biology* 2011, **12**(4):R41.
9. Yoo M, Shin J, Kim J, Ryall KA, Lee K, Lee S, Jeon M, Kang J, Tan AC: **DSigDB: drug signatures database for gene set analysis**. *Bioinformatics* 2015, **31**(18):3069-3071.
